# High-speed optical resolution photoacoustic microscopy with MEMS scanner using a novel and simple distortion correction method

**DOI:** 10.1038/s41598-022-12865-3

**Published:** 2022-06-02

**Authors:** Ryo Shintate, Takuro Ishii, Joongho Ahn, Jin Young Kim, Chulhong Kim, Yoshifumi Saijo

**Affiliations:** 1 Graduate School of Biomedical Engineering, Tohoku University, Sendai, 980-8579 Japan; 2 Frontier Research Institute for Interdisciplinary Sciences, Tohoku University, Sendai, 930-8555 Japan; 3 Department of Convergence IT Engineering, Electrical Engineering, and Mechanical Engineering, Pohang University of Science and Technology (POSTECH), Pohang, 37673 Republic of Korea

**Keywords:** Applied optics, Optical imaging, Ultrasound, Microscopy, Biomedical engineering, Mechanical engineering, Biophotonics, Photoacoustics

## Abstract

Optical resolution photoacoustic microscopy (OR-PAM) is a remarkable biomedical imaging technique that can selectively visualize microtissues with optical-dependent high resolution. However, traditional OR-PAM using mechanical stages provides slow imaging speed, making it difficult to biologically interpret in vivo tissue. In this study, we developed a high-speed OR-PAM using a recently commercialized MEMS mirror. This system (MEMS-OR-PAM) consists of a 1-axis MEMS mirror and a mechanical stage. Furthermore, this study proposes a novel calibration method that quickly removes the spatial distortion caused by fast MEMS scanning. The proposed calibration method can easily correct distortions caused by both the scan geometry of the MEMS mirror and its nonlinear motion by running an image sequence only once using a ruler target. The combination of MEMS-OR-PAM and distortion correction method was verified using three experiments: (1) leaf skeleton phantom imaging to test the distortion correction efficacy; (2) spatial resolution and depth of field (DOF) measurement for system performance; (3) in-vivo finger capillary imaging to verify their biomedical use. The results showed that the combination could achieve a high-speed (32 s in 2 × 4 mm) and high lateral resolution (~ 6 µm) imaging capability and precisely visualize the circulating structure of the finger capillaries.

## Introduction

Photoacoustic microscopy (PAM) is a typical implementation of photoacoustic imaging (PAI) and is an innovative biomedical imaging technology that achieves higher resolution, rich optical contrast, and superior penetration depth compared to optical imaging^1–3^. PAM systems employ a confocal structure of laser irradiation and ultrasound detection to generate and detect photoacoustic waves from an optical absorber in living tissues, and then perform volumetric data acquisition and image reconstruction of the subject. Furthermore, because the contrast of the reconstructed PA images depends on the properties of the optical absorber inside the tissue, PAM is capable of performing label-free anatomical and functional imaging using an endogenous absorber such as oxy/deoxy-hemoglobin^4^, melanin^5^, and DNA/RNA^6^. Owing to these unique advantages, PAM has been widely used in numerous clinical and preclinical studies, such as oncology^5,7^, neuroscience^8,9^, histology^10–13^, dermatology^14^ ophthalmology^15,16^, and cardiology^17^.

Optical resolution photoacoustic microscopy (OR-PAM) is a PAM implementation that achieves a higher spatial resolution^1,18,19^. It uses tightly focused laser irradiation with a much smaller focus area than that of the acoustic reception beam. As a result, OR-PAM has achieved an optical-dependent high lateral resolution of 0.2–10 µm with a penetration depth of up to 1 mm^20–24^, furthermore its high resolution indicates the potential to perform (1) clinical imaging of capillaries and their metabolic activity, (2) preclinical imaging of small animals such as mice^21,25^.

Despite their prominent imaging capability, conventional OR-PAM systems have a limited imaging speed because they employ a two-dimensional mechanical scanner equipped with stepping motor stages for volumetric PA data acquisition^22,23^. In particular, OR-PAM requires a large number of scan points to reflect its high lateral resolution for imaging quality; therefore, two-dimensional mechanical scanning requires a very long imaging time. In a previous paper, OR-PAM based on the mechanical stage had a 2D tomographic (B-mode) scan rate of 1 Hz/mm, and a 3D volumetric acquisition time of 7 min over a 1 × 1 mm^2^ area^23,26,27^. The disadvantage of long imaging times of conventional OR-PAM not only causes motion artifacts during PA imaging of living tissue, but also makes it difficult to biologically interpret the obtained PA images. Therefore, techniques that enable an increase in the imaging speed of OR-PAM are in high demand.

In earlier attempts at high-speed OR-PAM, a fast laser scanning method with a galvanometer scanner was used^28–30^. Fast laser scanning was performed, and the generated PA waves were detected using a fixed ultrasound transducer with an unfocused or poorly focused detection spot, however, since the laser irradiation and ultrasound detection were not confocal, this configuration provided a low SNR. In addition, it had a very limited field of view (FOV) within the focal spot size of ultrasound detection. Here, the SNR in this scanning method was low because the fixed ultrasound transducer causes wave interference and wavefront distortion when receiving PA waves generated by a source at axial and lateral offsets from the focal point^31–33^. This resulted in distortion of the signals acquired outside the focal region, causing deviation of frequency content from the detectable bandwidth and sensitivity, degrading the SNR. Specifically, Seeger et al. measured the spatial characterization (total impulse response) of fixed ultrasound transducer by stepwise translating the transducer along the axial direction from the focal position toward negative/positive offset and lateral direction gradually away from the central axis of the transducer to analyze the spatial dependency of detected impulse responses^33^. They verified that the axial offset reduces frequency sensitivity, and the lateral offset causes both waveform distortion and significant degradation in frequency sensitivity (especially in the high-frequency components). This resulted in degradation of detection sensitivity, image distortion, and spatial resolution during imaging.

In contrast, the latest generation of fast OR-PAM employs a waterproof MEMS scanning mirror^34–43^. In the fast PAM system, the coaxially and confocally adjusted laser irradiation beam and the ultrasound reception beam were scanned together by the MEMS mirror placed in the water. Hence, high-speed imaging was achieved while maintaining a high SNR and a better FOV. For example, Kim et al. showed that waterproof-MEMS mirrors scanning both the optical excitation and ultrasound detection (50 MHz center frequency) achieved SNR of 39 dB, a significant improvement over that of the conventional laser scanning method with fixed ultrasound transducer (SNR of 25 dB)^37^. They also achieved an FOV of 6.6 mm within the maximum scanning angles of the MEMS mirror, which was superior than the FOV of the conventional laser-scanning PAM using fixed transducer with a similar frequency of 40 MHz (Unfocused: 300 µm/Focused: 30–70 µm)^29^. The superior sensitivity and field of view of MEMS scanning PAM made it suitable for biological applications. In addition, the MEMS scanning mirror has recently been commercialized^44–46^. The abovementioned novel actuator has the potential to effectively overcome the problem of the imaging speed of conventional OR-PAM and enhance its impact in the fields of medical and life sciences. Therefore, in this study, we aimed to develop a high-speed OR-PAM (MEMS-OR-PAM) system that utilizes a commercialized 1-Axis MEMS mirror.

**Figure 1:**
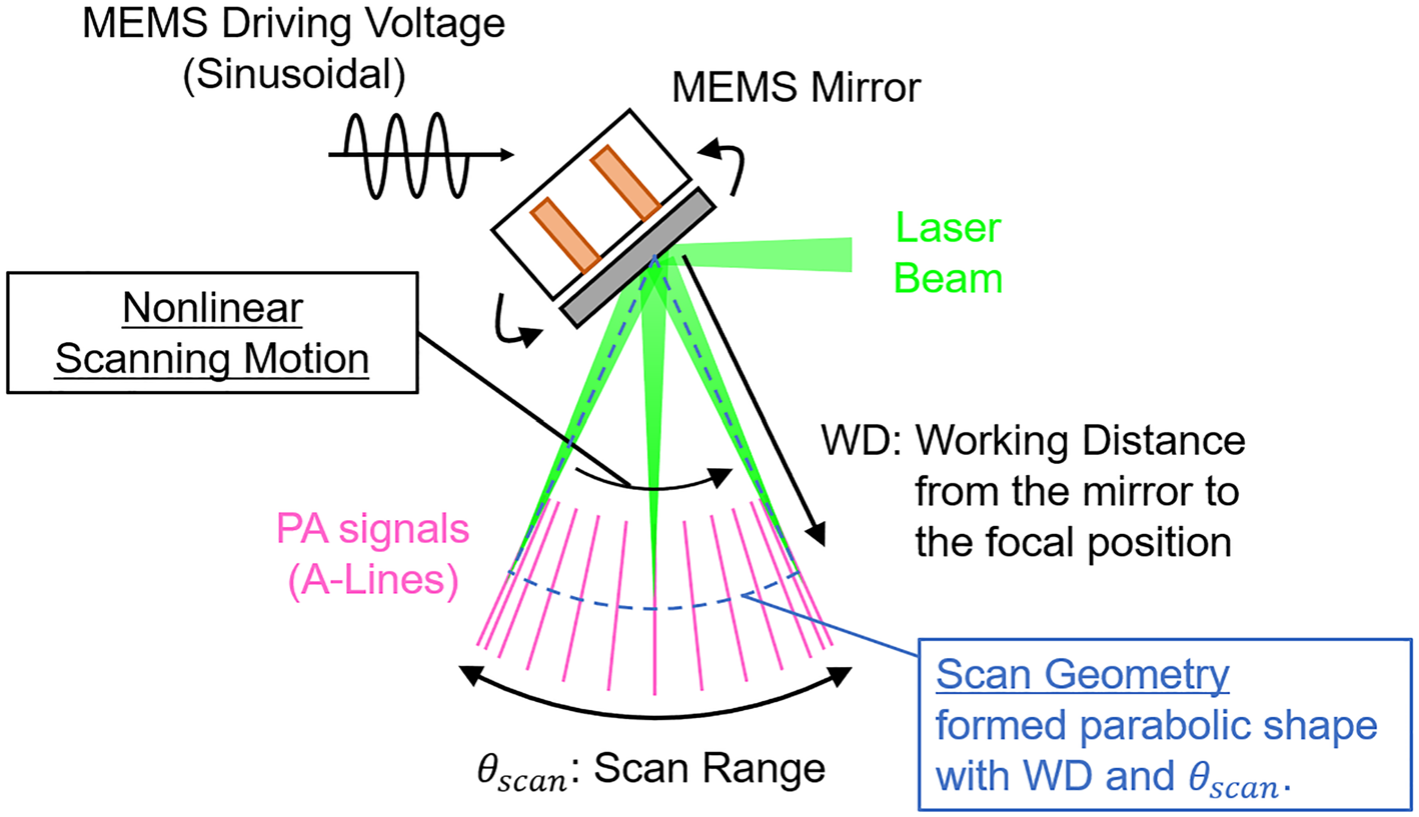
Scan geometry and the nonlinear motion of MEMS scanning mirror.

While performing high-speed imaging with a MEMS scanning mirror, as shown in Fig. 1, the scan geometry and the nonlinear scanning motion of the MEMS mirror must be considered as it causes distortion in the obtained volumetric PA data and the reconstructed PA images^39,47^. The nonlinear scanning motion of the MEMS mirror is caused by the sinusoidal driving voltage when the mirror is operated at a high speed. Thus, it can be corrected by considering the sinusoidal driving characteristics of the scanning angle. In addition, the scan geometry of the MEMS mirror has a parabolic shape formed by the range of the scan angle (${\theta }_{scan}$) and the working distance (WD) from the center of rotation of the mirror to the focal point of the confocal opto-acoustic beam. Therefore, it is considered as the polar coordinate, and the coordinate transformation can correct the image distortion caused by the geometry. However, it should be noted that accurate definition of the geometry requires to have the prior knowledge of the following two parameters: (1) WD is related to not only the focal length of the opto-acoustic beams but also the positional relationship of elements such as the MEMS mirror installed in the focused beam, and (2) ${\theta }_{scan}$ related to the amplitude of the MEMS driving voltage. The most typical method for determining these parameters involve performing multiple calibration measurements with the MEMS-OR-PAM system under various conditions^36,38^. However, determination of accurate parameters is time-consuming and complicated, making it difficult to correct the distortions in practical imaging situations. For example, ${\theta }_{scan}$ changes its characteristics in a complex way depending on the amplitude and frequency of the driving voltage, and the mechanical property depends on the surrounding temperature, resulting in extensive measurements to cover all measurement conditions and understand their characteristics. Typically, more than 30 measurements were required to determine the characteristics of ${\theta }_{scan}$ of the 1-axis MEMS mirror which consists of voltage-scan angle property and driving frequency-scanning angle property, which could be highly time-consuming^37,39^. In addition, the WD complicatedly varies depending not only on the focal length of the opto-acoustic coaxial beam, but also on the positional relationships of the elements installed on the coaxial beam, such as the beam combiner and the MEMS mirror^34,35^. Therefore, measuring the experimental value of WD was challenging. Therefore, we needed a method to quickly estimate these two parameters (WD, ${\theta }_{scan}$) and the geometry, and easily correct the image distortion caused by the MEMS mirror without performing complicated experiments.

Therefore, in this research, in addition to the development of a novel MEMS-OR-PAM system, we propose a novel method that can easily correct the spatial distortion caused by high-speed scanning of a MEMS mirror by calibrating the system using a micron-scale ruler. Briefly, the procedure consisted of five steps.

1. Calibrate the PA imaging to achieve the desired imaging range by referring to the scale of the ruler.
2. Set virtual WD and ${\theta }_{scan}$ to virtually define the polar coordinate geometry for the acquired volumetric PA data.
3. Introduce the conversion equation to consider the nonlinear scan motion of the MEMS mirror into the volumetric PA data and then perform the coordinate transformation.
4. Evaluate the effect of distortion correction from the reconstructed image; and
5. By repeating steps (2)–(4), determine the robust WD, ${\theta }_{scan}$, and scan geometry to achieve the most distortion-free image.

Unlike the previous methods, the calibration method is required to run the imaging sequence only once before imaging the actual subjects (e.g., phantoms and living tissue). The effectiveness of the developed MEMS-OR-PAM and the calibration method were evaluated by leaf skeleton phantom imaging, spatial resolution and depth of field (DOF) measurements, and finger capillary imaging.

## Materials and methods

### Single-axis waterproof MEMS (1A-WP-MEMS) scanning mirror

This study employed a single-axis waterproof MEMS scanning mirror (1A-WP-MEMS, OptichoMS-001, Opticho Inc. Ltd., Republic of Korea). The fabrication method and specifications of the 1A-WP-MEMS mirror were described in a previous paper^37^, and they have been adopted in many studies^34,36,38,39,44–46^. Briefly, the MEMS scanner consisted of two major modules: a “movable structure layer” and “fixed block of electromagnets”. The movable structure layer contained a PDMS layer, an acrylic frame, an aluminum mirror (reflection rate in water: 92% in laser/84% in ultrasound), and neodymium magnets. The PDMS layer and acrylic frame firmly prevented the electromagnet of the fixed block from leaking electricity, thus forming a waterproof structure for the MEMS scanner. When a driving voltage is applied to the electromagnets, a magnetic field is generated, thus generating an attractive and repulsive force between the electromagnets and the neodymium magnet. This phenomenon generates a torque in the mirror on the movable layer, resulting rotation of the mirror to scan the laser and ultrasound coaxial beams. Here, the available optical spectral range for excitation is from visible to infrared because the waterproof MEMS mirror was coated with aluminum which has a high optical reflection rate of 90% in the spectral range. In case it is water-immersed, its reflectance would be lower in an infrared range due to high optical absorption on water. We considered 532 nm wavelength, where the effect of water on reflectance is relatively small, and it has over 90% reflectance. The resonant frequency of the mirror used in this study was approximately 60 Hz (in air)/45 Hz (in water), and the maximum scan angle range was approximately 18°.

### Experimental setup of MEMS-OR-PAM

**Figure 2:**
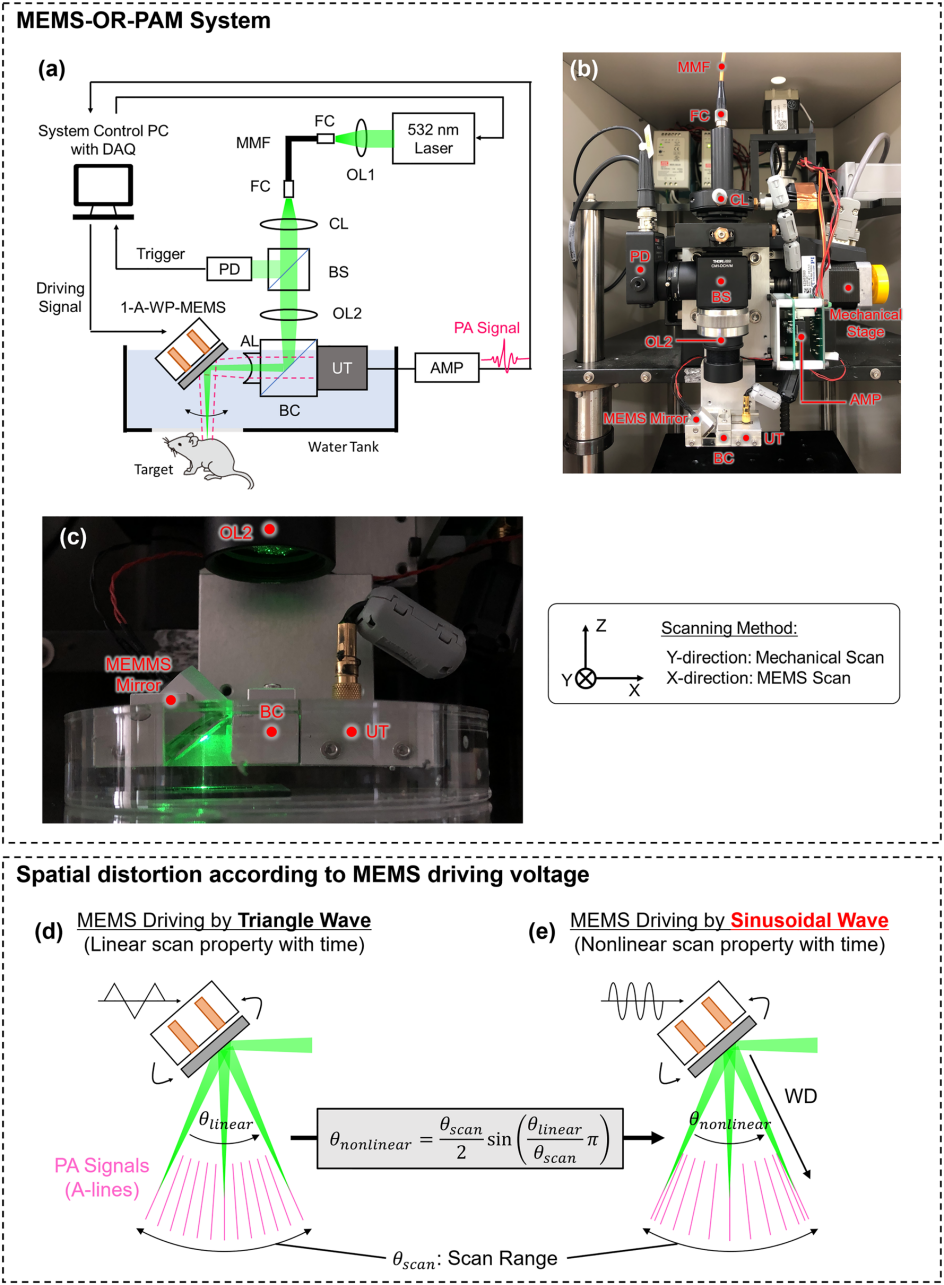
Schematic of experimental setup (upper dotted square) and spatial distortion according to MEMS driving voltage (lower dotted square). (**a**) Block diagram of developed MEMS-OR-PAM system. (**b**) Photo image. (**c**) Close-up view of the scanning part. (**d**) MEMS driving by triangle wave (linear with laser PRF timing). (**e**) MEMS driving by sinusoidal wave (nonlinear with laser PRF timing).

Figure 2a–c shows a schematic diagram and photographs of the high-speed MEMS-OR-PAM system developed in this study. In this system, an Nd:YAG 532 nm laser (AWAVE532-1W-10K, Advanced Optowave; 10 ns pulse width; 10–100 kHz pulse repetition rate) was employed as the light source. The emitted laser beam was conveniently guided through the objective lens (OL1) to the PAM system by coupling to a multimode fiber (FC, FG010LDA, Thorlabs; 10 µm core diameter; 0.1 NA). The output laser beam from the fiber was shaped by a collimating lens (CL) and then split into two beams using a beam splitter (BS, FM01R; Thorlabs; > 85% transmission rate). One beam (< 15%) was reflected and guided to a photodetector (PD, PDA36A-EC, Thorlabs) as a trigger signal for data acquisition timing. The other beam (> 85%), which was reshaped to a diameter of ~ 10 mm, was tightly focused by an achromatic doublet lens (OL2, AC254-060-A, Thorlabs; 60 mm focal length; 0.2 NA). The focused laser beam was then combined coaxially and confocally with an acoustic reception beam using an opto-acoustic beam combiner (BC), an unfocused ultrasound transducer (UT, V214-BC-RM: 50 MHz center frequency: 80% − 6 dB bandwidth, Olympus NDT), and an acoustic lens (AL, NT45-010, Edmund, USA; 0.25 NA). The BC consisted of a correction lens (NT67-147, Edmund, USA), an aluminum-coated prism (MRA10-G01, Thorlabs, USA), and an uncoated prism (PS910, Thorlabs, USA).All these components were joined together using an optical adhesive (NOA61). This confocal structure was applied to maximize the SNRs of the PA signals. The opto-acoustic beam was reflected by the 1A-WP-MEMS mirror and irradiated onto the imaging target to generate the PA waves. The PA waves were detected by the ultrasound transducer and then amplified using two amplifiers connected in series (AMP, ZX60-3018G-S + Mini-Circuit: 46 dB total gain). A high-speed digitizer (ATS9350, Alazar Technologies: 500 MS/s sampling frequency) was used to record the PA signals.

High-speed scanning was performed using a sinusoidal driving voltage applied to the MEMS mirror. The scanning sequence was performed using a combination of a high-speed MEMS scan in the X-direction and a constant-velocity linear motion of the mechanical stage in the Y-direction (see detailed scanning sequence in Supplementary Text A, Fig. S1). This scanning sequence was constructed based on the theory used in previous research on high-speed PAM^35,43,48^. As an overview of the scanning sequence, the PA data acquisition in the X-direction was performed during the half cycle of the MEMS scan, controlled by the driving frequency. The data acquisition in the Y-direction was performed synchronously with that in the X-direction. Thus, the speed of the mechanical stage was adjusted to move in the Y-direction with one step during the half cycle of the MEMS scan. For example, when imaging in the interest area of 2 × 2 mm with a step size of 400 × 400 points (5 µm averaged step width) using a 10 kHz laser PRF, MEMS scanning speed has 12.5 Hz frequency (80 ms in period), velocity of the mechanical stage in Y-direction is 62.5 µm/s, and the time for a 3D volumetric PA data acquisition is estimated to be 32 s. Even if more scanning points (smaller step width) are used to consider a spatial Nyquist, the scan speed can still be set as high by setting higher laser PRF.

### Signal processing and image reconstruction

The acquired volumetric PA signals were converted to analytic signal data by applying an IIR bandpass filter (5–120 MHz) and Hilbert transform, which was followed by the reconstruction of a 2D cross-sectional image by applying the Maximum Intensity Projection (MIP; thereafter called as C-mode) method to the volume PA envelopes^49^. In this study, a C-mode image shows the projected top-view (X–Y plane) of the imaging subject, while the B-mode image shows a slice of side views (X–Z or Y–Z planes) of the acquired three-dimensional image data. These image reconstructions were performed in real time using LabVIEW software (National Instruments) and post-processed with MATLAB 2021b (MathWorks).

It is important to note that the volumetric PA data acquired and reconstructed at this stage involve spatial distortions caused by fast MEMS scanning. Spatial distortion was corrected using the method described in the next section.

### Correction method for the distortion derived by MEMS mirror scanning

As described in the introduction, owing to the nonlinear scanning motion and the polar coordinate scan geometry of the mirror, the volumetric PA data acquired with a high-speed MEMS-OR-PAM system are spatially distorted^39^. The nonlinear scanning motion of the MEMS mirror is caused by the MEMS driving voltage, which is applied as a sinusoidal wave for high-speed operation.

Here, when the driving voltage is applied as a triangular wave, as shown in Fig. 2d, the scanning property changes linearly with time (= ${\theta }_{linear}$). In such a case, the volumetric PA data acquired at the timing of the laser pulse repetition frequency (PRF) can be treated as a simple polar coordinate. However, the so-called quasi-static scanning with triangle or sawtooth driving voltage required high frequency with high-order harmonics (typically 6–7th order or higher), which compromised the accuracy of the mirror scan in high-speed operation^50^. Therefore, to perform stable and accurate scanning in quasi-static MEMS scan, it was usually necessary to limit the driving frequency to less than 1/6–1/7 of the resonance frequency to accommodate such harmonics, resulting in slow scanning speed which was not suitable for real-time imaging. Using a complex controller was possible a solution for stable quasi-static scanning while maintaining the scan quality and fast scanning speed^51^, but it required the complexity of the MEMS-OR-PAM control system, which was not suitable for clinical applications.

On the other hand, when the MEMS driving voltage is applied as a sinusoidal wave, as shown in Fig. 2e, high-speed operation can be achieved as the actuator can move at a frequency closer to its resonant frequency. In previous research, high-speed PAM system using sinusoidal driving voltage achieved high-speed scanning at a B-scan rate of 400 Hz^35^, which was more than eight times faster than the conventional MEMS-OR-PAM using triangular driving voltage (5–50 Hz in B-scan rate)^34^. In addition to the scan speed, sinusoidal driving MEMS system does not require a complicated controller for driving voltage control, which allows it to introduce high-speed imaging at low cost. These advantages of stability, fast speed, and low cost are significant for applying the high-speed OR-PAM system for clinical use. However, as a trade-off for the imaging speed, the scanning angle transitions nonlinearly with time (= ${\theta }_{nonlinear}$). The nonlinear scanning angle characteristics when the MEMS mirror is driven by a sinusoidal wave can be expressed by the following equation^47^:

1 $${\theta }_{nonlinear}=\frac{{\theta }_{scan}}{2}sin \left(\frac{{\theta }_{linear}}{{\theta }_{scan}}\pi \right),$$

where ${\theta }_{nonlinear}$ is the nonlinear scanning angle characteristics, ${\theta }_{scan}$ is the range of the scan angle in the imaging, and ${\theta }_{linear}$ is the linear scanning property assumed by the PA acquisition timing with the laser PRF.

**Figure 3:**
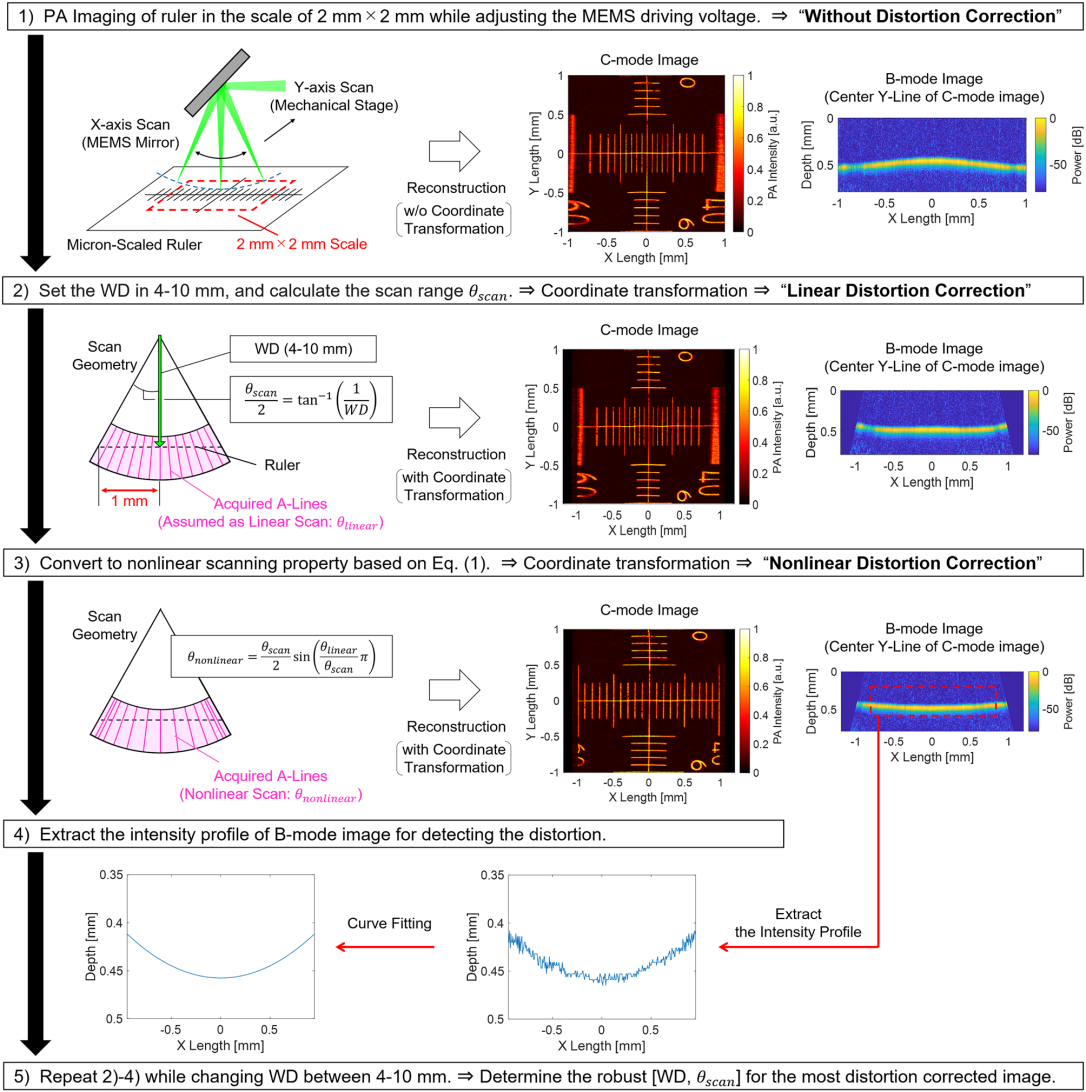
Proposed calibration method for the distortion correction using a ruler.

The polar coordinate geometry in the MEMS scan can be determined by the working distance (WD) between the MEMS mirror and the focal position of the confocal opto-acoustic beam and the range of the scan angle (${\theta }_{scan}$). In general, since the relationship between ${\theta }_{scan}$ and the applied driving voltage amplitude can change under several parameters, such as the scan speed and mechanical properties of the MEMS mirror, obtaining prior knowledge of ${\theta }_{scan}$ is difficult^36,37,39^. In addition, WD cannot be simply determined because it is affected by the following factors: (1) the experimental focal length of the opto-acoustic beam, and (2) the positional relationship of elements such as MEMS mirrors and beam combiners installed on the path of the focus beam^34,35^. To estimate these parameters just before running imaging of the actual subject without any time-consuming or complex procedure and easily correct its spatial distortion, a calibration method based on a single imaging of a ruler-target was proposed. The outline of the proposed method is shown in Fig. 3, which consisted of the following five steps.

1. PA imaging of the ruler (0.1 mm Eyepiece Micrometer, Muhwa eCommerce Co. Ltd, Div. x: 0.1 mm; y: 0.1 mm) under the same imaging conditions as the subsequent phantom or tissue imaging. Here, we set the imaging area to X × Y = 2 × 2 mm with 400 × 400 points and a laser PRF of 10 kHz. The depth position of the ruler was adjusted to the opto-acoustic focus. During the imaging, real-time image reconstruction (C-mode and B-mode image without distortion correction) was performed using LabVIEW, and the amplitude of the sinusoidal MEMS driving voltage was adjusted to include the ruler’s scale of the imaging region of interest into the C-mode image. In this way, the optimum MEMS driving voltage amplitude for an imaging area of interest can be easily determined.
2. *Linear distortion correction* To define the polar coordinate geometry, WD and ${\theta }_{scan}$ were set virtually. WD can be selected from the range of 4–10 mm, which is a visually determined rough value. Using the virtual WD, the scan range ${\theta }_{scan}$ can be calculated as follows:
  2 $${\theta }_{scan}=2{\mathrm{tan}}^{-1}\left(\frac{{X}_{scale}/2}{WD}\right)=2{\mathrm{tan}}^{-1}\left(\frac{1}{WD}\right)$$
  where ${X}_{scale}$ is the imaging range of interest in the X-direction (2 mm). The polar coordinate geometry can be defined by virtually setting WD and ${\theta }_{scan}$. However, as mentioned earlier, the acquisition timing of the A-lines is based on the laser PRF, thus the scanning angle property was assumed to be linear in this step. Therefore, if the Cartesian coordinate transformation is performed with post-processing in this step, the reconstructed image would still remain distorted in both the C- and B-mode images.
3. *Nonlinear distortion correction* Applying Eq. (1) for transformation to the nonlinear scanning angle property. The resulting nonlinear polar coordinate geometry was transformed into Cartesian coordinates to generate the distortion-free reconstructed image.
4. To detect the slight image distortion of the B-mode image, the intensity profile of the B-mode image was extracted and fitted to detect the flatness of the profile. This process considered the fact that the ruler target has a flat surface sputtered on a flat glass substrate.
5. The steps (2)–(4) are repeated while changing the virtual setting value of WD between 4 and 10 mm. From the reconstructed C- and B-mode images and the flatness of the intensity profile of B-mode, we determined the best conditions of WD and ${\theta }_{scan}$ to obtain the most distortion-free polar coordinates.

The polar coordinates obtained by the above method are also applied for image distortion correction in PA imaging of phantoms or living tissues under similar imaging conditions.

### Imaging of a leaf skelton phantom

To verify whether the proposed distortion correction method with a ruler and the obtained polar coordinates are also practical for imaging other measurement targets, PA imaging of a black-stained leaf skeleton phantom was performed. The phantom was attached to the bottom of a dish filled with water and placed on the opto-acoustic beam focus of the MEMS-OR-PAM. PA imaging was conducted in the region, X × Y = 2 × 6 mm, with 400 × 1200 points. The laser PRF was 10 kHz, and the emitted energy was ~ 100 nJ/pulse. After PA imaging, the obtained 3D volumetric data were applied to the linear/nonlinear distortion correction in the X-direction (MEMS scan direction) by using the polar coordinate map obtained by the proposed method with a ruler. Then, the C-mode image and its depth projection image were reconstructed using the volumetric PA data before and after distortion correction. The reconstructed PA images were compared with the photo image of the phantom to verify the effectiveness of the distortion correction.

### Spatial resolution and DOF measurement

To verify whether the developed MEMS-OR-PAM along with the calibration method for distortion correction has sufficient spatial resolution for microtissue imaging, we performed lateral/axial resolution and DOF measurements.

For lateral resolution measurement, PA imaging was performed using the USAF1951 target. The measurement range was X × Y = 2 × 2 mm (800 × 800 points), the laser PRF was 10 kHz, and the laser energy was < 100 nJ/pulse. USAF1951 has microline patterns sputtered on a glass substrate. The lateral resolution conversion value was calculated from the smallest line patterns that could be separated in the C-mode image after nonlinear distortion correction. In addition, to improve the reliability of the measurements, we also applied the Line Spread Function (LSF) method in this study. Specifically, the LSF method was performed in the following three steps: (1) extract the edge spread function (ESF) of the line pattern in the C-mode image, (2) calculate the LSF, which is the differential waveform of the ESF, and (3) measure the lateral resolution by calculating the full width at half maximum (FWHM) of the LSF.

**Figure 4:**
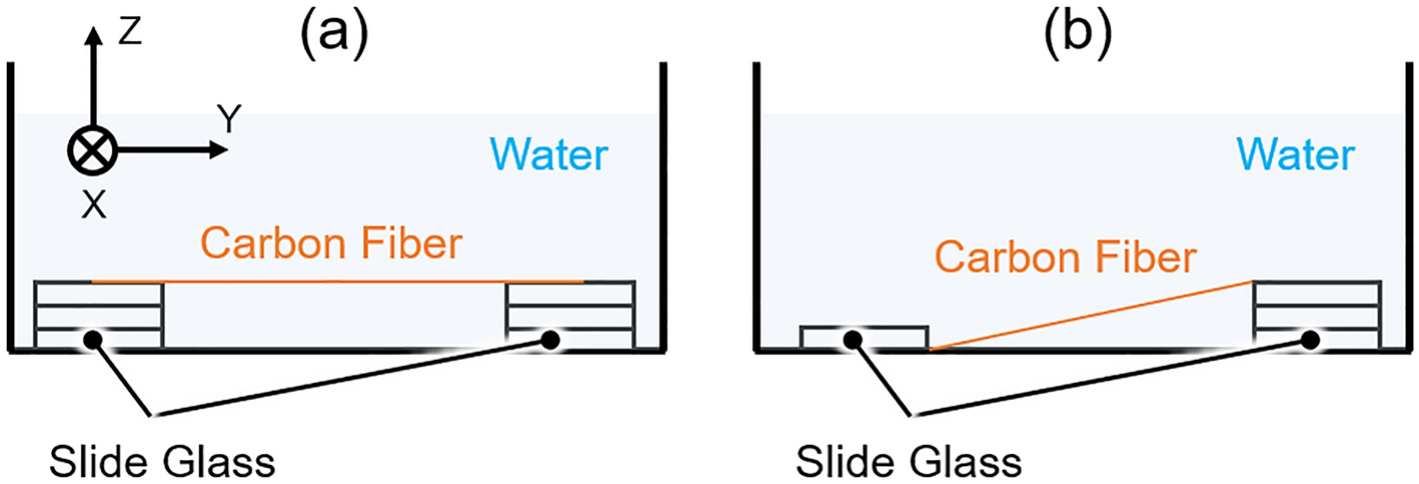
Schematic for carbon fiber preparation. (**a**) Horizontally stretched for axial resolution measurement. (**b**) Vertically tilted for DOF measurement.

For the axial resolution measurement, PA imaging was performed with a horizontally stretched carbon fiber with a diameter of 7 µm, as shown in Fig. 4a. The measurement range was X × Y = 2 × 1 mm (800 × 400 points), the laser PRF was 10 kHz, and the laser energy was < 100 nJ/pulse. The obtained 3D volumetric data were subjected to nonlinear distortion correction, and then the C- and B-mode images were reconstructed. The axial resolution was then calculated from the FWHM of the intensity profile in the B-mode image.

For the DOF measurement, a vertically tilted carbon fiber with a diameter of 7 µm was used as the imaging target, as shown in Fig. 4b. PA imaging of the target was performed in the range of X × Y = 2 × 10 mm (800 × 4000 points), the laser PRF was 10 kHz, and the laser energy was < 100 nJ/pulse. The obtained 3D volumetric data were subjected to nonlinear distortion correction followed by reconstruction of the C-mode and depth projection images. Subsequently, the profiles of the maximum PA intensity and FWHM of the fiber along the depth were created by associating both images. From these profiles, the DOF was evaluated by two indicators: the depth range where (1) PA intensity was half the peak, and (2) FWHM was expanded by $\sqrt{2}-1$ of the lateral resolution from the minimum FWHM. These indicators were used in a previous study^52^, and the FWHM was associated with the theoretical evaluation^53^. Furthermore, in this measurement, the center frequency and bandwidth of the PA signals (impulse responses) from different depths generated from the fiber were calculated to evaluate the distance-dependency of imaging sensitivity.

In addition to the measurements of the spatial resolution and DOF, their theoretical values were calculated (described in Supplementary Text B, Fig. S2). As a result, the theoretical values of lateral resolution, axial resolution, and DOF were found to be 7.0, 32.6, and 165.3 µm, respectively.

### In-vivo microvascular imaging of human finger

To verify the effectiveness of the MEMS-OR-PAM with distortion correction for in vivo imaging, PA imaging of the fingertip microvasculature was performed. All experimental procedures followed a protocol approved by the Institutional Review Board (IRB) at the Graduate School of Engineering, Tohoku University. All methods were performed in accordance with the relevant guidelines and regulations. A healthy volunteer provided fully informed consent for the imaging of his finger. In the imaging, the measurement range was X × Y = 2 × 4 mm (400 × 800 points), the laser PRF was set to 20 kHz, and the pulse energy was 400 nJ/pulse. Here, because the laser PRF is twice as high as the previous in vitro measurements, the driving frequency of the MEMS mirror was set to be twice as fast as before. Thus, resulting in a slight change in the characteristics of the driving voltage amplitude and the corresponding scan range. Therefore, calibration was performed again using a ruler to adjust the MEMS driving voltage before commencing in vivo PA imaging, followed by microvascular imaging. After the imaging, C-mode images without distortion correction and with nonlinear distortion correction were reconstructed, respectively. In addition, a B-mode image after nonlinear distortion correction was reconstructed to investigate the penetration depth in in vivo imaging.

Before imaging, the laser safety on the skin surface was calculated (Supplementary Text C, Fig. S3). As a result, in this experiment in which the focus position was set to the penetration depth of 300 µm where the capillary is located, the fluence on the skin surface was calculated to be 13.7 mJ/cm^2^, which met ANSI safety standards^54^.

## Results

### Distortion correction

Using the developed MEMS-OR-PAM system, the proposed distortion correction method using a ruler target was successfully validated. As described in Fig. 3, the calibration process was repeated by changing WD from 4 to 10 mm in 1 mm steps to find the robust pair of WD and ${\theta }_{scan}$. The observed distortions at each tested WD value are shown in Supplementary Movie 1.

**Figure 5:**
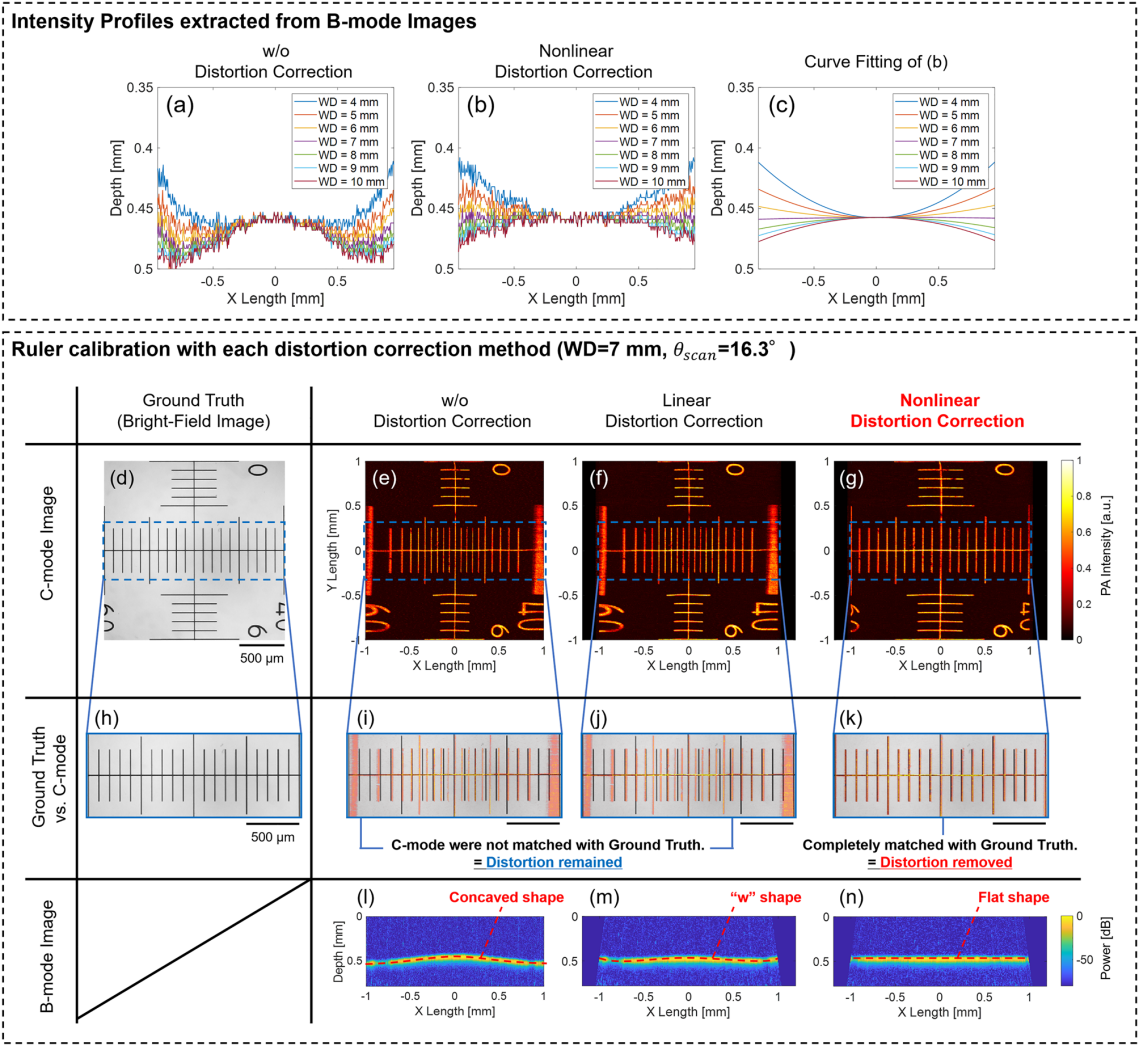
*Upper dotted square* Comparison of the intensity profile extracted from the B-mode image with (**a**) linear distortion correction, (**b**) nonlinear distortion correction, and (**c**) curve fitting result of (**b**). *Lower dotted square* Results of ruler calibration with each distortion correction method in WD = 7 mm, ${\theta }_{scan}$=16.3°: C-mode (Upper), enlarged C-mode (Middle, for comparing with the ground truth), and B-mode (Lower, about a center Y-line in C-mode) images. (**d,h**) Ground truth (Bright-field image and enlarged image). (**e,i,l**) PA images without distortion correction. (**f,j,m**) PA images with linear distortion correction. (**g,k,n**) PA images with nonlinear distortion correction.

Figure 5a–c show the PA intensity profiles extracted from the B-mode ruler images during the distortion correction process. In Fig. 5a, the intensity profile after linear distortion correction, a nonlinear shape occurred in every WD because the nonlinear scan property was not considered. On the other hand, Fig. 5b shows the intensity profile after nonlinear distortion correction, and the nonlinear shape was removed, and only the curved shape of the profile was confirmed. The shape was close to horizontal when WD was in the 6 mm to 8 mm range. Figure 5c shows the results of the curve fitting of the profile in Fig. 5b, which could clarify the profiles and showed that the profile became the most horizontal and completely removed the distortion at WD = 7 mm. The scan range at WD = 7 mm was calculated to be ${\theta }_{scan}$= 16.3° from Eq. (2).

Figure 5d–n shows the results of the reconstructed PA images without/with distortion correction in [WD, ${\theta }_{scan}$] = [7 mm, 16.3°]. Here, Fig. 5d shows the bright-field image of the ruler, prepared as the ground truth to compare the effect of the distortion corrections. Figure 5e–g show the C-mode images in each distortion correction method. In Fig. 5e, the result without distortion correction, since the image reconstruction was conducted from the volumetric PA data treated as Cartesian coordinates, the reconstructed image was distorted by both the scan geometry and nonlinear motion of the MEMS scanning. In Fig. 5f, the results of the linear distortion correction, the C-mode image was still distorted owing to the nonlinear motion even after considering the polar coordinate geometry of the MEMS scanning. Significantly, in the C-mode image, the central part in the X-direction had compressed distortion, and the outer region had extended distortion. This behavior was consistent with the nonlinear scanning property of the MEMS mirror shown in Fig. 2a. However, Fig. 5g, the results of the nonlinear distortion correction, clearly show that the image distortion could be removed, and the ruler’s scale was reproduced at equal intervals by eliminating the effect of the nonlinear motion of the MEMS scanning. Additionally, Fig. 5i–k show the enlarged C-mode image to compare with the ground truth shown in Fig. 5h, which highlights the effect of the distortion correction. From these results, in Fig. 5g, the C-mode image after the nonlinear distortion correction was fully matched with the ground truth, which indicated the complete elimination of distortion.

Figure 5l–n show the B-mode images in each distortion correction method extracted from the centerline of the ruler’s scale. Figure 5l, the B-mode image without distortion correction, the intensity profile of the ruler, which should be reproduced flat, was visualized as a curved shape. Moreover, in Fig. 5m, the result of distortion correction, the curvature was suppressed because a polar geometry was considered, but the nonlinear profile (“w” shape) remained uneliminated. Therefore, these two correction methods were not suitable for the optimal reconstruction of B-mode images due to the lack of consideration of either or both scan geometry and nonlinear scanning characteristics. However, the result with nonlinear distortion correction in Fig. 5n, which consider both the polar geometry and the nonlinear scanning characteristics, visualized the profile as flat. Therefore, nonlinear distortion correction with the ruler calibration also showed the availability for distortion removal in the reconstruction of the B-mode images.

In the above-described results, we compared distortion correction approaches based on the assumption that the MEMS mirror geometry is used. Therefore, distortion correction approach of applying a Cartesian coordinate geometry with a nonlinear scanning property was excluded. Here, all distortion correction approaches, including the distortion correction approach, can be compared in Supplementary Fig. S4. From the figure, in case of applying the distortion correction of Cartesian scan geometry with nonlinear scan property, it could also correct the distortion in the C-mode image as well as nonlinear distortion correction, compared to the images without distortion correction and with linear distortion correction. However, in the B-mode image, the approach could not correct the distortion and produced a curved profile of the ruler because the MEMS mirror’s original scan geometry was not considered. Thus, the distortion correction using a Cartesian scan geometry with nonlinear scan property could not correctly reconstruct the 3D structures of the imaging targets. Therefore, to correctly recover the 3D structure of such objects, the scan geometry of the MEMS mirror must be correctly defined. In this regard, the nonlinear distortion correction shown in Fig. 5g,k,n could correctly estimate the scan geometry as well as the nonlinear scan property, and thus, it could perform distortion correction most effectively.

From these results, the proposed distortion correction method with a ruler suggested that it could readily determine the polar coordinate geometry of MEMS-OR-PAM and provide distortion-free reconstructed images after nonlinear distortion correction.

### Leaf skelton phantom imaging

**Figure 6:**
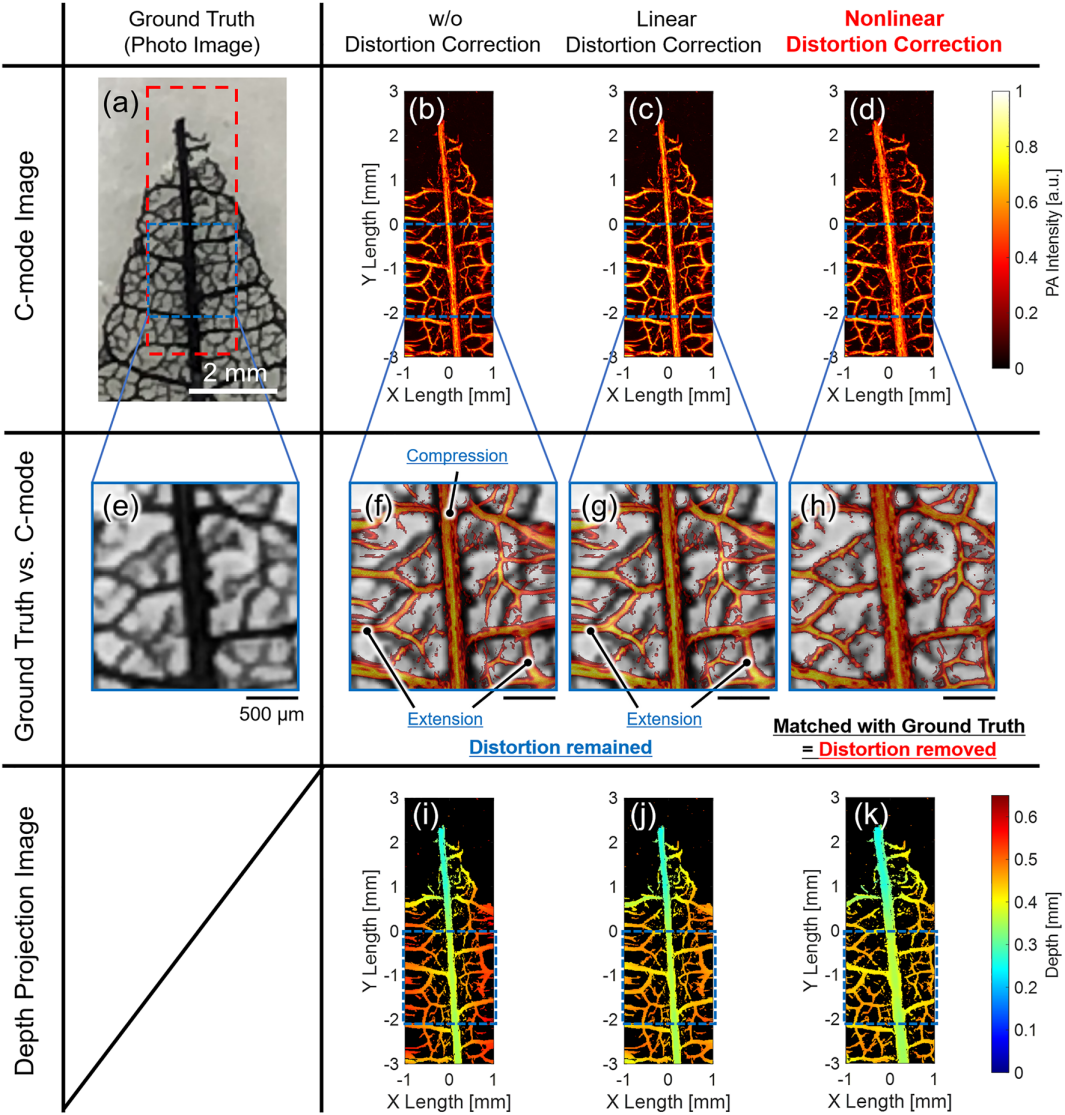
Results of leaf phantom PA imaging with C-mode image (Upper), enlarged image along the blue dotted square (Middle, for comparing with the ground truth), and depth projection image (Lower). (**a,e**) Ground truth (Photo image and the enlarged image). (**b,f,i**) PA images without distortion correction. (**c,g,j**) PA images with linear distortion correction. (**d,h,k**) PA images with nonlinear distortion correction.

Figure 6 shows the PA imaging results of the leaf Skelton phantom, whose photo image is shown in Fig. 6a. While Fig. 6b,f,i do not show any distortion correction, Fig. 6c,g,j and Fig. 6d,h,k show C-mode images, their enlarged C-mode images for comparison with enlarged Photo image (ground truth) in Fig. 6e, and the depth projection images after applying linear and nonlinear distortion corrections, respectively.

For the C-mode images, the results in Fig. 6b,c, which are the images without and with linear distortion correction, respectively, showed that the phantom’s structures were not reconstructed correctly and were distorted compared to the photo image in Fig. 6a. Additionally, the corresponding enlarged C-mode images in Fig. 6f,g clearly show the distortions in the X-direction (center region: compression/outer region: extension) owing to the nonlinear MEMS scanning motion, resulting in a mismatch with the enlarged photo image in Fig. 6e, which was prepared as the ground truth. On the other hand, as shown in Fig. 6d,h, which were the C-mode image and its enlarged image with the nonlinear distortion correction, respectively, the distortions were removed, and completely matched with the ground truth. Therefore, in the reconstruction of C-mode image, the phantom's structure was correctly reconstructed by proposed nonlinear distortion.

For the depth projection image, the depth range of the reconstructed phantom was the narrowest in Fig. 6k with the nonlinear distortion correction, and it qualitatively showed that the distortion in the depth direction was removed. To quantitatively evaluate the distortion as the imaging flatness, the maximum and minimum depth values and the depth width were calculated for the blue dotted square in Fig. 6i–k, as shown in Table 1. As a result, the depth range with nonlinear distortion correction was the smallest at 183 µm (Max: 519 µm/Min: 336 µm), which was 12.9% narrower than the result without distortion correction (210 µm) and 28.2% better than the result after linear distortion correction (255 µm). This result presents the effect of distortion correction as the imaging flatness.

**Table 1:** Comparison of the depth range of the phantom.

	Depth image		
	Without distortion correction (μm)	Linear distortion correction (μm)	Nonlinear distortion correction (μm)
Max depth	591	546	519
Min depth	336	336	336
Depth range	255	210	183

These qualitative and quantitative evaluations showed that the proposed distortion correction method using a ruler and the obtained nonlinear scan geometry works well for imaging other objects such as phantoms.

### Spatial resolution and DOF measurement

**Figure 7:**
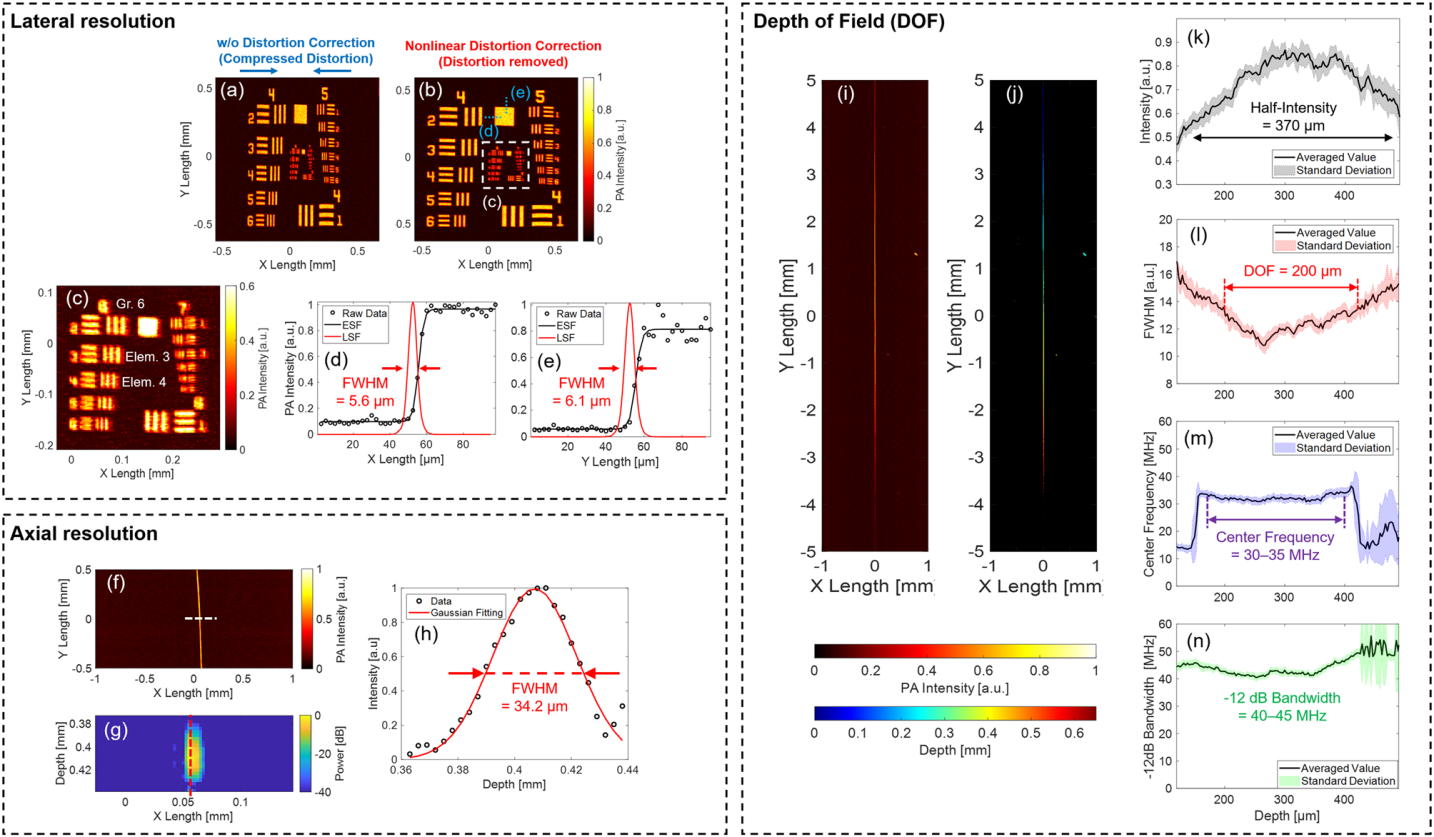
Results of spatial resolution and DOF measurements. *Upper dotted square* Lateral resolution measurement result with USAF 1951 target. (**a**) C-mode image without distortion correction. (**b**) C-mode image with nonlinear distortion correction. (**c**) Enlarged C-mode image along the white dotted square in (**b**). (**d**) Result of LSF method along the X-direction blue dotted line in (**b**). (**e**) Results of the LSF method along the Y-direction blue dotted line in (**b**). *Middle dotted square* Axial resolution measurement result with a horizontally stretched carbon fiber. (**f**) C-mode image with nonlinear distortion correction. (**g**) B-mode image about the white dotted line in (**a**). (**h**) PA envelope profile along the red dotted line in (**b**), and Gaussian fitting. *Lower dotted square* DOF measurement result with a vertically tilted carbon fiber. (**i**) C-mode image with nonlinear distortion correction. (**j**) Depth projection image. (**k**) PA intensity of the fiber along the depth. (**l**) FWHM of the fiber along the depth. (**m**) Center frequency of the PA signals along the depth. (**n**) Frequency bandwidth (− 12 dB) of the PA signals along the depth.

Figure 7a–e shows the results of the lateral resolution measurement using USAF1951. The lateral resolution calculation was performed for the C-mode image with nonlinear distortion correction, as shown in Fig. 7b. From its enlarged C-mode image with a nonlinear distortion correction shown in Fig. 7c, the smallest line pattern that could be visibly separated in both the X- and Y-directions was around Group 6 Element 3–4. Thus, the conversion value of lateral resolution was calculated as 5.5–6.2 µm. In addition, in Fig. 7d,e, the ESFs in the X-and Y-directions were extracted, and the LSFs and their FWHMs were measured. As a result, the FWHMs were calculated as X: 5.6 µm/Y: 6.1 µm. Therefore, the experimental lateral resolutions from the two methods were consistent, and thus the results were highly reliable. Thus, the lateral resolution of the developed MEMS-OR-PAM was experimentally determined to be ~ 6 µm, which was very close to the theoretical value (7.0 µm). This high lateral resolution shows the potential to sufficiently visualize microtissues, such as human capillaries.

Figure 7f–h shows the results of axial resolution measurements using horizontally stretched carbon fibers. In Fig. 7h, the PA intensity profile in the depth direction along the red dotted line in Fig. 7g was extracted, fitted by a Gaussian function, and then the FWHM of the fitted curve was utilized as the axial resolution of the MEMS-OR-PAM system. As a result, the experimental axial resolution was measured as 34.2 µm, which was in good agreement with the theoretical value (32.6 µm) calculated by the bandwidth of the ultrasound transducer.

Figure 7i–n shows the results of the DOF measurement using a vertically tilted carbon fiber. In the images in Fig. 7i,j, the fiber was vertically tilted along the Y-direction. Therefore, as shown in the C-mode image of Fig. 7i, in the central region of the Y-direction, where the fiber was within focus, the imaging contrast and sharpness were excellent, whereas in the outside of the Y-direction, where the fiber was out of focus, the imaging contrast and sharpness were found to be worse. In the depth projection image of Fig. 7j, the depth was calculated from the reception delay time of the PA intensity of Fig. 7i with intensity-based threshold cutting to project only the depth information from the fiber selectively. Based on these two images, the PA intensity and FWHM of the fiber along the depth direction were calculated, as shown in Fig. 7k,l, respectively. From the result of Fig. 7k, the depth range at which the PA intensity is half of the peak was calculated to be 370 µm. In Fig. 7l, the FWHM of the carbon fiber was calculated to be in range 11–16 µm with a depth range of 300 µm (150–450 µm). The minimum FWHM (11 µm) was slightly larger than the diameter of the fiber (7 µm) because the true profile of the fiber was convolved with the point spread function (PSF) of imaging system, which is based on the experimental lateral resolution. The measured value of DOF was calculated as the depth range when the FWHM is 13.4 µm (11 µm + 2.4 µm), which is the value when the FWHM is expanded by $\sqrt{2}-1$ of the lateral resolution (6 µm) from the minimum FWHM. As a result, the measured DOF was calculated as 200 µm, which was slightly larger than the theoretical value (165.3 µm). Figure 7m,n show the center frequency and bandwidth (− 12 dB) of the PA signal (impulse response) at the maximum intensity in each depth shown in Fig. 7k. These two frequency indicators could maintain high/wide sensitivities in the wide depth range of 150–400 µm: 30–35 MHz (center frequency) and 40–45 MHz (− 12 dB bandwidth). In the center frequency shown in Fig. 7m, detection performance declined beyond the depths range of 150–400 µm because the PA intensity drops below half of the peak, as shown in Fig. 7k. On the other hand, the frequency bandwidth shown in Fig. 7n could detect without the effect of PA intensity degradation because it detected down to 1/4 (− 12 dB) of the peak PA intensity, achieving good sensitivity over a wide depth range. Therefore, the developed MEMS-OR-PAM was shown to have the potential to visualize microtissues while maintaining high contrast, lateral resolution, and imaging sensitivity within a long depth range.

Therefore, the developed MEMS-OR-PAM was shown to have the potential to visualize microtissues while maintaining high contrast and lateral resolution within a long depth range.

### In-vivo microvascular imaging of human finger

**Figure 8:**
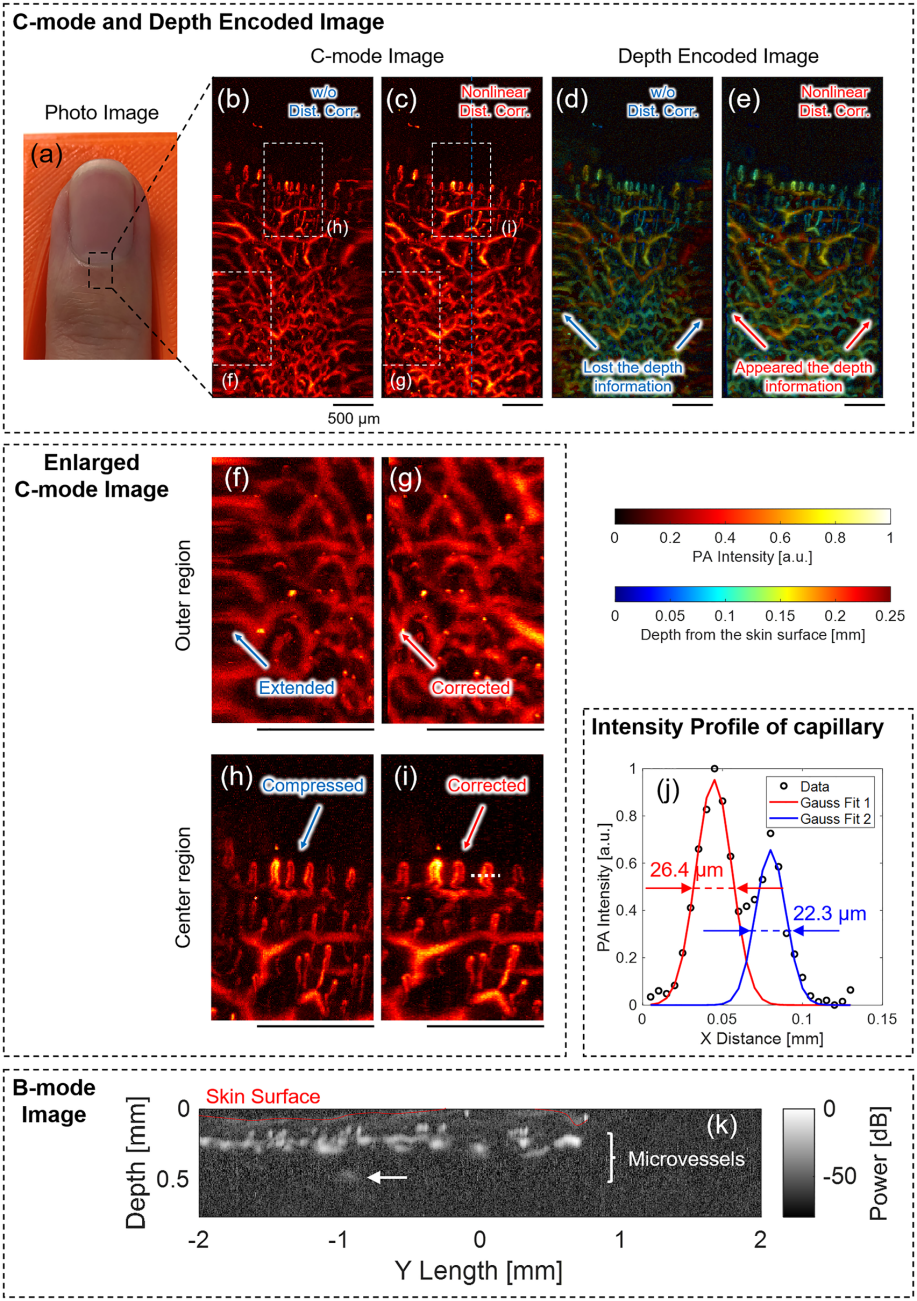
Result of microvascular imaging of human finger. (**a**) Photo image. (**b,c**) C-mode image: (**b**) without distortion correction, and (**c**) with nonlinear distortion correction. (**d,e**) Depth encoded image: (**d**) without distortion correction, and (**e**) with nonlinear distortion correction. (**f,g**) Enlarged C-mode image in the outer region of (**b,c**): (**f**) without distortion correction and (**g**) with nonlinear distortion correction. (**h,i**) Enlarged C-mode image in the center region of (**b,c**): (**h**) without distortion correction and (**i**) with nonlinear distortion correction. (**j**) PA intensity profile along the white dotted line in (**i**). (**k**) B-mode image along the blue dotted line in (**c**).

Figure 8 shows the results of the microvascular PA imaging of the fingertips. The measurement time for the PA volumetric data acquisition was 32 s.

Figure 8a is a photo image of the fingertip, and the PA imaging was performed in the black dotted square. Figure 8b,c show the C-mode images reconstructed without distortion correction and with nonlinear distortion correction, respectively. From the two results, unlike the C-mode image without distortion correction shown in Fig. 8b, the nonlinear distortion correction shown in Fig. 8c indicates that the image distortion was successfully removed. To confirm the effect of the distortion correction in more detail, Fig. 8f–i show the enlarged C-mode images cut along the white dotted squares in Fig. 8b,c, respectively. In Fig. 8f,g, the outer region of the C-mode images, extended distortion was removed after the nonlinear distortion correction in Fig. 8g. Furthermore, in Fig. 8h,i, the center region of the C-mode images, compressed distortion was corrected after nonlinear distortion correction in Fig. 8i. Therefore, the nonlinear distortion correction demonstrated that it could visualize the entire microstructure of the microvessels, including the capillaries and their unique circulatory structures.

Figure 8d,e show the depth encoded image corresponding to the C-mode images in Fig. 8b,c. In Fig. 8d, depth information, especially in the outer region, was lost because the depth encode image determined the depth projection location based on the intensity from the vessels in the C-mode image, and depth projection was difficult in the outer region where the vascular structure was poorly reproduced in C-mode. However, Fig. 8e, the results after nonlinear distortion correction, depth information of the microvessels was clearly visualized in the entire image because the vessel structures were correctly reproduced in the C-mode image of Fig. 8c. These results showed that nonlinear distortion correction could accurately visualize the three-dimensional structure of microvessels.

Figure 8j shows the PA intensity profile extracted from the white dotted line in Fig. 8i, and the profiles distinguished the two blood vessels for the round trip in the circulatory structure. By applying the Gaussian fitting and calculating the FWHM of the intensity profile, the diameters of the two blood vessels were found to be 26.4 and 22.3 µm. In addition, the FWHMs of the five circulatory structures (10 microvessels) in Fig. 8i were also calculated and averaged to obtain accurate measurement values of the diameter. As a result, the average diameter of the capillaries was 25.1 µm (SD 4.1 µm), and this result was within the range of 10–35 µm, which is the diameter of previously reported human capillaries^55,56^.

Figure 8k shows the B-mode image along the blue dotted line in Fig. 8c. From this result, it can be concluded that microvessels mainly exist at a depth range of 200–300 µm from the skin surface, and as shown by the white arrow, the deepest microvessel was visualized at a depth of ~ 500 µm from the skin surface. Therefore, the in vivo penetration depth achieved by MEMS-OR-PAM was at least 500 µm.

## Discussion

### Usefulness of the proposed distortion correction method

The distortion correction method using a ruler calibration, which was proposed in this study, could: (1) quickly adjust the MEMS driving voltage according to the imaging area of interest (scan range) by referring to the ruler’s microscale (takes only 10 min to adjust), (2) easily estimate the scan geometry and nonlinear scan motion of the MEMS scanning by post-processing, which previously required complicated premeasurement to estimate, and (3) precisely correct the spatial distortion. As a result, the MEMS-OR-PAM using the distortion correction method could accurately visualize the capillary structure, as shown in Fig. 8c. Here, the remarkable point is that it has even visualized the circulatory structure of human capillaries, which was almost the first achievement of fast OR-PAM. Although a few studies have previously visualized the structure by employing conventional mechanical scanning OR-PAM^57^, its slow imaging speed made it difficult for biological interpretation. On the other hand, MEMS-OR-PAM provides fast imaging speed, however, despite being capable of clearly visualizing the capillaries of nude mice, separately visualize the circulatory structure of human finger capillaries, many studies found it challenging to separately visualize the circulatory structure of human finger capillaries^25,43,58^. This is because the human skin has strong optical scatterers such as melanin, which degrades the lateral resolution to slightly blur the circulatory structure; thus, the additional image distortion leads to difficulty in separately visualizing such blurred structures. Therefore, for such near-clinical subjects, the distortion correction accuracy of the MEMS-OR-PAM is directly linked to the visualization performance of the circulation structure. The proposed calibration method using a ruler performed precise distortion correction based on an accurate estimation of the MEMS scan geometry. Therefore, it could be concluded that the MEMS-OR-PAM system in this study improved the separation performance of the circulatory structure of the capillaries. Such accurate PA imaging can promote the biological interpretation of microtissues, such as capillaries, and accurately grasp morphological changes, such as structural abnormalities of capillaries^59^. To this degree, the biomedical applications of MEMS-OR-PAM method can be improved by combining it with the proposed distortion correction.

Furthermore, the advantage of the proposed distortion correction method is its flexibility and simplicity. Even if the measurement conditions are changed, the distortion correction can be executed quickly by calibrating the ruler again. For example, even in a case similar to that of the finger microvascular imaging shown in Fig. 8, where: (1) the laser PRF was changed to 20 kHz, which is higher than the phantom measurements (10 kHz), and (2) the characteristics between the MEMS driving voltage amplitude and the scan angle were slightly changed, the drive voltage corresponding to the experimental conditions can be obtained, and the distortion correction can work again by only performing Step 1) of the ruler calibration method in Fig. 3 assuming that the MEMS scan range (2 mm) in imaging is the same. Such flexibility of the proposed distortion correction may facilitate the medical application of MEMS-OR-PAM.

### Imaging speed and FOV of MEMS-OR-PAM

Imaging speed and FOV are essential factors when performing in vivo imaging with MEMS-OR-PAM. In this study, in vivo finger microvascular imaging was performed in the range of X × Y = 2 × 4 mm in 32 s (4 s in 1 mm^2^) with B-scan rate of 25 Hz. It was 105 times faster than the typical mechanical scanning OR-PAM (7 min in 1 mm^2^)^23^. This real-time imaging of MEMS-OR-PAM greatly prevents the occurrence of motion artifacts during bioimaging. As proof of this, capillary imaging using the developed MEMS-OR-PAM, as shown in Fig. 8c, clearly visualized the circulatory structure of capillaries, which should not be visible even if a small amount of motion artefacts occur. However, to visualize a more dynamic microvascular structure in the living body and the flow of red blood cells inside the microvessels through it in real time, it is necessary to have a wider FOV and faster imaging.

To improve the imaging speed, employing a higher PRF of the laser is the most effective solution, assuming that the imaging range and number of scan points are constant. However, it is a prerequisite that the PA waves generated for each laser pulse do not interfere with each other in the living body^39^. Assuming that the speed of sound of living tissue is 1540 m/s and the maximum penetration depth is 500 µm, the propagation time for PA waves to travel from the maximum depth of the tissue to the surface is 325 ns. In this case, the maximum effective PRF of the laser was 3 MHz. Therefore, high-speed imaging within the PRF range of 10–100 kHz, which is the operating range of the laser used in this study, can be performed without any problems. In case of using a PRF faster than 100 kHz as the laser source, it is necessary to consider the influence of the pulse energy and SNR of the PA signals, which are reduced by trade-off, and the limitation of the physical limitation of the operating speed of the MEMS mirror. In addition, we can refer to previous studies on high-speed PAM systems using high-speed laser PRFs to achieve faster imaging. For example, Chen et al. constructed a high-speed PAM system with a laser PRF of 200 kHz for real-time visualization of microvascular dynamics in the mouse’s ear, and imaged it within a scan range of 1.5 mm (500 pixels) at a B-scan rate of 400 Hz^60^. Yao et al. developed a high-speed functional photoacoustic microscopy with a laser PRF of 500 kHz for 3D high-resolution, high-speed imaging of mouse brain, and achieved a scan range of ~ 3 mm (1250 pixels) at a B-scan rate of 400 Hz^35^. In addition, Kim et al. developed high-speed OR-PAM with a water-immersible galvanometer scanner and a laser PRF of 500 kHz for visualizing capillaries in mouse ears and brain and human fingers, and achieved real-time imaging within a scan range of 2.4 mm (500 pixels) at a B-scan rate of 500 Hz^25^. Thus, by increasing the laser PRF to several hundred kHz, developed MEMS-OR-PAM achieves more than ten times faster imaging rate while maintaining the FOV, which has the potential for real-time monitoring of microvascular dynamics.

Regarding the improvement of imaging FOV, we must consider each in the X- and Y-directions because the developed MEMS-OR-PAM employs a different scan method in each direction (X: MEMS scan, Y: Mechanical scan). The FOV in the Y-direction was determined by the operating length of the mechanical stage. The DOF measurement shown in Fig. 7i–l confirmed that the mechanical stage could operate in the range of 10 mm with no problems. In addition, the maximum operating length of the mechanical stage used in this study was defined as 20 mm, which was sufficient for in vivo microtissue imaging in a large FOV in the Y-direction. On the other hand, the FOV in the X-direction is determined by the physically limited scan angle (18°) of the MEMS mirror. In this study, the scan angle for the imaging range of 2 mm was estimated to be 16.3°, which is close to the limitation. Therefore, when only using the MEMS mirror, the FOV in the X-direction is limited to a smaller area than the mechanical scan. To overcome this limitation of FOV in the X-direction, a combination of the MEMS mirror and a mechanical stage is required^38^.

### Imaging fidelity in non-uniform spatial sampling

In the developed MEMS-OR-PAM system, high-speed imaging was achieved by a hybrid scan method that applies nonlinear high-speed scanning with a MEMS mirror in the X-direction and constant-velocity linear motion of the mechanical stage in the Y-direction. However, the combination of these two methods had a problem of spatially non-uniform sampling in the nonlinear MEMS scan and skewed sampling positions along the mechanical-moving axis. Therefore, the effect of such non-uniform spatial sampling on the image fidelity needs to be well discussed. To discuss these issues, we considered using the imaging results of in-vivo human finger capillaries.

To verify the imaging fidelity by the non-uniform spatial sampling in human finger capillary PA imaging shown in Fig. 8, we calculated the variation of the step width in the X-direction when MEMS scanning was performed in 2 mm with 400 points (5 µm average step width), as shown in Supplementary Fig. S5. The scanning property of the MEMS mirror changed nonlinearly (sinusoidal) with a range of 2 mm (− 1 mm to + 1 mm) as shown in Fig. S5a, resulting in the step width varying along the sampling point, as shown in Fig. S5b. The maximum step width was 7.8 µm in time, which was ~ 1.6 times larger than the averaged step width of 5 µm. However, in this study, we processed a spatial resampling (linear interpolation) when performing a Cartesian coordinate transformation of the non-linear polar coordinate. Therefore, the step width after the coordinate transformation was virtually uniformed as 5 µm, reducing the effect of temporal variations in step width. Consequently, in the imaging of human capillaries with the average diameter of 25 µm, the fidelity of the imaging results was not affected, because the imaging target was much larger than the theoretical lateral resolution (7 µm) and the virtual step width (5 µm). Additionally, the skewed sampling positions along the mechanical-moving axis were almost negligible. In this study, we used a hybrid scanning with constant velocity linear motion of the mechanical stage in the Y-direction during MEMS scanning in the X-direction, and the sequence was executed so that the Y-direction moved one step (5 µm) during one-line MEMS scan in the X-direction (The details of the scanning sequence are described in Supplementary Text A, Fig. S1). Therefore, the average moving distance in the Y-direction for each pixel in the X-direction was calculated as 12.5 nm, which was 1/2000 smaller than the average diameter of capillaries and almost negligible for the fidelity of the imaging results. These verifications indicated that the spatial sampling variability in the X- and Y-direction scans was almost negligible in terms of image fidelity for our target capillary imaging.

### Effect of changing thickness of scan volume during MEMS scanning on impulse response and spatial resolution

In MEMS scanning, the distance between the beam focus and the imaging target varies with the scan geometry. If the distance is larger than the imaging DOF, the received signal’s sensitivity, bandwidth, shape, and impulse response can be significantly degraded, as reported in previous studies^33^. The change in impulse response is expected to significantly affect the imaging sensitivity and spatial resolution, thereby affecting the accuracy of the proposed distortion-corrected image. Therefore, we performed an additional analysis on the variation of impulse response and spatial resolution due to the change of the distance in the thickness direction when scanning MEMS in the range of X = 2 mm by the following theoretical and practical measurements.

**Figure 9:**
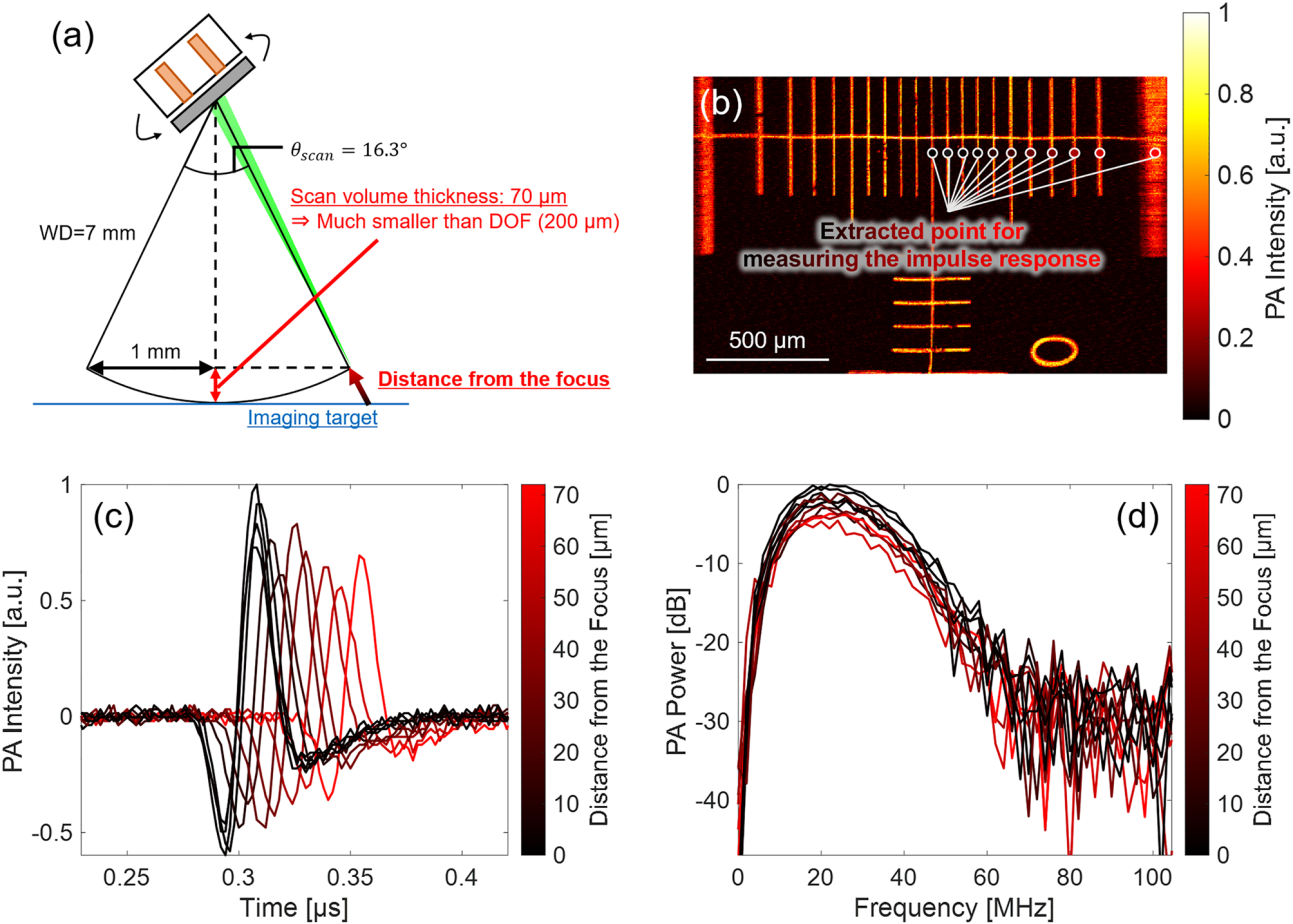
Investigation of the spatial variation of signal detection sensitivity derived by MEMS scanning. (**a**) MEMS scan geometry in X = 2 mm, which represents the variation of the focal position in the thickness direction (Max: 0.07 mm) was within the experimental DOF (0.20 mm). (**b**) C-mode image (w/o distortion correction) of the ruler indicating the extracted position for measuring the signal impulse response. (**c**) Time waveforms of the impulse responses. (**d**) Frequency waveforms of the impulse responses.

First, Fig. 9 shows the theoretical and experimental validation results of the spatial imaging sensitivity of the MEMS scan. As shown in Fig. 9a, in the scan geometry with WD = 7 mm and ${\theta }_{scan}$=16.3°, the scan volume thickness was calculated as approximately 70 µm, which was sufficiently smaller than the experimental DOF (200 µm) in Fig. 7l. Therefore, it was predicted that the spatial resolution and imaging sensitivity would be maintained in the MEMS scan range of 2 mm. To verify this prediction, we extracted PA signals from 11 positions of the ruler’s C-mode image (w/o distortion correction) at different distances to the beam focus, shown in Fig. 9b, and compared their extracted impulse responses. Figure 9c compared the time waveforms of the impulse responses which shows delays in the received signals depending on the distance between the target and the beam focus. However, the differences of peak intensities were approximately 60% along with the distances, which indicated the excellent sensitivity maintained within the MEMS scanning range. Figure 9d compared the frequency waveforms of the impulse responses, which confirmed that the peaks of the spectrums varied by 5–6 dB along with the distances, but the shape of the spectrum was almost unchanged. These results demonstrated that the MEMS scanning was conducted within the DOF, resulting in the imaging sensitivity being maintained at a good level. Even if the scan volume thickness becomes more significant than 70 μm, the distance-dependency of the impulse response (intensity, center frequency, and frequency bandwidth) can be quantitatively determined from the results of DOF measurement shown in Fig. 7k,m,n. These results showed that the sensitivity was well maintained at least within ± 100 μm of the beam focus.

**Figure 10:**
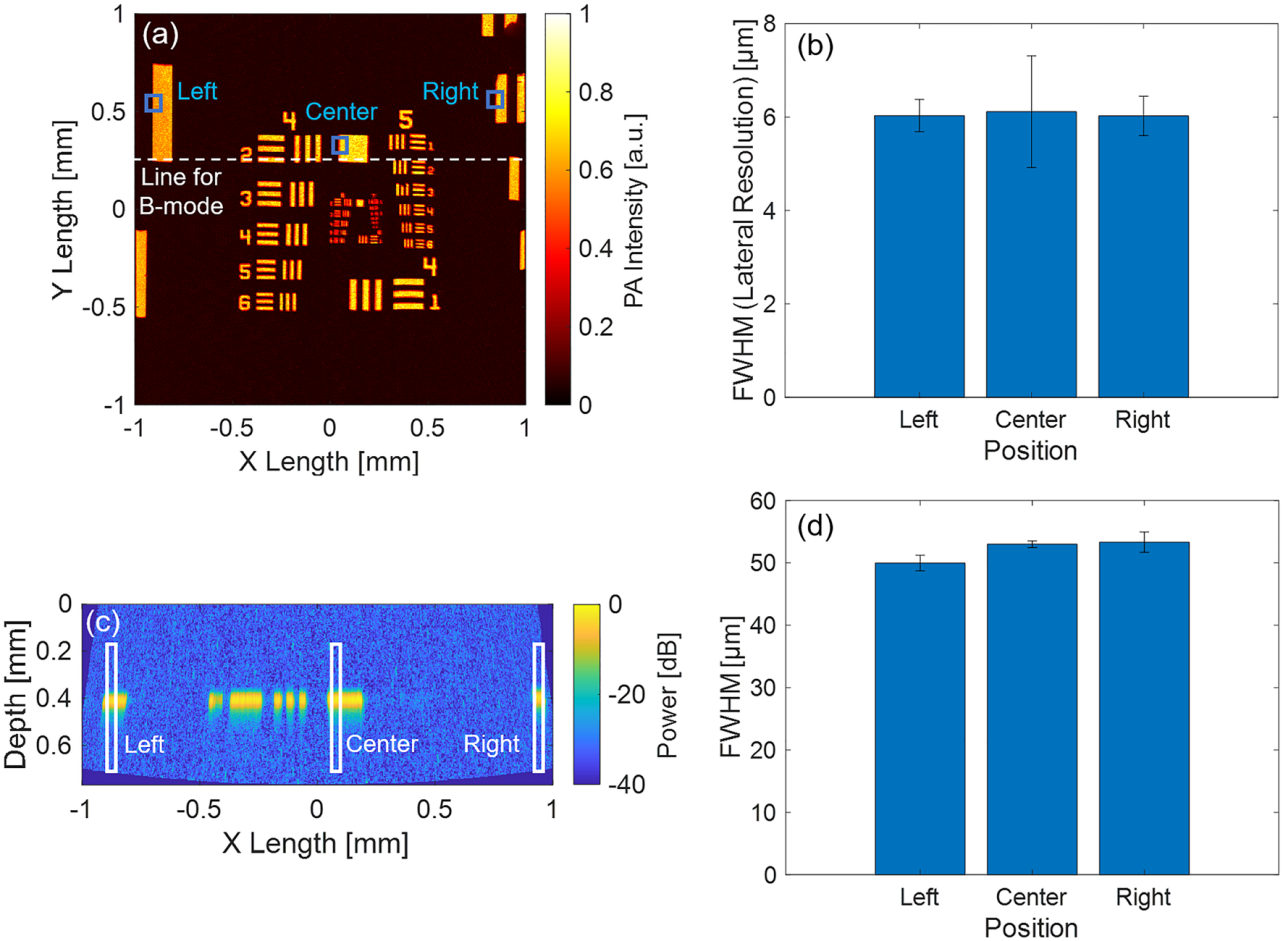
Investigation of the spatial variation of lateral/axial resolution using USAF1951 target. (**a**) C-mode image (nonlinear distortion correction, X × Y = 2 × 2 mm with 800 × 800 points, 10 kHz PRF). (**b**) Lateral resolutions in the position of left/center/right of (**a**). (**c**) B-mode image (nonlinear distortion correction). (**d**) FWHMs of the intensity profiles in the position of left/center/right of (**c**).

To investigate the effect of the superior sensitivity in the FOV on the spatial resolution, we measured the variation of the spatial resolution at the left, center, and right end of the MEMS scan direction using the results of the USAF1951’s C- and B-mode images shown in Fig. 10a,c. Figure 10b shows the measured spatial variation of the averaged lateral resolution at the left, center, and right end of Fig. 10a, which were 6.0 ± 0.7 µm/6.1 ± 2.4 µm/6.0 ± 0.8 µm, respectively. This demonstrated that high lateral resolution was maintained within the FOV. Figure 10d shows the averaged FWHM of the intensity profiles at the left, center, and right end of B-mode image shown in Fig. 10c, which were 50.0 ± 2.5 µm/53.0 ± 1.1 µm/53.3 ± 3.3 µm, respectively. The averaged FWHM differed from the experimental axial resolution of 34.2 µm in Fig. 7h, because the test target was a large sample which was not considered as a point source for the axial resolution measurement. Therefore, we focused only on the differences between the average FWHM of each position. The results suggested that the FWHM varied only approximately 3 µm (6%) within the FOV and that the axial resolution hardly changed within the FOV.

These results indicated that the developed system could perform MEMS scans within a thickness variation much smaller than DOF of the MEMS-OR-PAM, thus maintaining the sensitivity of the impulse response and maintaining high spatial resolution within the imaging FOV. We believe that it significantly contributed to the accurate distortion reduction in the proposed distortion correction method.

### Medical application of MEMS-OR-PAM

One of the practical medical applications of MEMS-OR-PAM is the evaluation of peripheral vascular disease caused by impaired microcirculation. For example, it has been suggested that thinning of capillaries contributes to hypertension^61,62^. Therefore, it can be used as a diagnostic index for subsequent diseases such as arteriosclerosis, myocardial infarction, and cerebral infarction. The developed MEMS-OR-PAM and the proposed distortion correction method have demonstrated the ability to visualize the structure of the capillaries selectively and clearly. Thus, making it possible to be used as a diagnostic technique for medical needs.

Furthermore, in the future, we plan to employ a two-wavelength laser source to visualize the distribution of oxy/deoxy-hemoglobin in the capillary. This would enable the developed MEMS-OR-PAM method to not only clearly visualize the circulating structure of capillaries but also monitor the oxygen metabolism between capillaries and the surrounding cells based on the spectral unmixing method^63^, thus making it possible to achieve functional PA imaging of microtissues and quantify the functional hemodynamics. When multi-wavelength lasers are added to the system, functional information at each measurement point can typically be acquired by irradiating these lasers at the same PRF and switching them one pulse at a time^64^. Therefore, imaging speed in multi-wavelength MEMS-OR-PAM is limited by the minimum switching time, which corresponds to 325 ns at a penetration depth of 500 µm. Thus, when dual-wavelength laser (e.g., 532 nm and 559 nm) is employed in developed MEMS-OR-PAM, there is no problem in performing high-speed imaging within the maximum PRF of 1.5 MHz for each laser.

## Conclusions

In this paper, we report the development of a high-speed OR-PAM system using a commercialized 1A-WP-MEMS scanning mirror and a novel calibration method that uses a micron-scale ruler to correct the spatial distortion caused by fast MEMS scanning. This novel method can easily and quickly find the scan geometry and nonlinear scan motion of MEMS mirrors without the need for complicated experimental measurements and can be applied for distortion correction. The combination of the MEMS-OR-PAM and the calibration method for distortion correction was verified using artificial and biological subjects, and the experimental results showed that the new fast OR-PAM system provides high-speed and high lateral-resolution imaging capabilities and precisely visualizes the circulating structure of capillaries in the human fingertip.

## Supplementary Information

Supplementary Information.

Supplementary Movie 1.
