# Supplementary material for: High-speed optical resolution photoacoustic microscopy with MEMS scanner using a novel and simple distortion correction method
Source: Sci Rep. 2022 Jun 2;12:9221. doi: 10.1038/s41598-022-12865-3 (PMC9163157; doi:10.1038/s41598-022-12865-3)
Supplement: Supplementary file 1 — Supplementary Information. [file 41598_2022_12865_MOESM1_ESM.docx]

Supplementary Information for

**High-Speed Optical Resolution Photoacoustic Microscopy with MEMS Scanner using a Novel and Simple Distortion Correction Method**

Ryo Shintate^1*^, Takuro Ishii^1,2^, Joongho Ahn^3^,

Jin Young Kim^3^, Chulhong Kim^3^, and Yoshifumi Saijo^1^

^1^*Graduate School of Biomedical Engineering, Tohoku University, Sendai 980-8579, Japan*

^2^*Frontier Research Institute for Interdisciplinary Sciences, Tohoku University, Sendai, Japan 930-8555, Japan*

*^3^Department of Convergence IT Engineering, Electrical Engineering, and Mechanical Engineering, Pohang University of Science and Technology (POSTECH), Pohang, 37673, Republic of Korea*

^*^Corresponding author. Email: [ryo.shintate.r4@dc.tohoku.ac.jp](mailto:ryo.shintate.r4@dc.tohoku.ac.jp), [saijo@tohoku.ac.jp](mailto:saijo@tohoku.ac.jp)

**This file includes:**

**Supplementary Text**

- **A. Scanning Sequence of MEMS-OR-PAM System**
- **B.** **Theoretical Spatial resolution and Depth of Focus (DOF)**
- **C. Laser Safety in Microvascular Imaging In-Vivo**

**Supplementary Fig. S1** Scanning sequence of MEMS-OR-PAM system.

**Supplementary Fig. S2** Simple optical focusing model of MEMS-OR-PAM. **Supplementary Fig. S3** Laser irradiation model for living tissue.

**Supplementary Fig. S4** Comparison of all distortion correction approaches in ruler imaging.

**Supplementary Fig. S5** Scanning characteristics of a MEMS scan in the range of X＝2 mm at 400 points.: (a) Position in the X direction. (b) Property of the step width.

**Movie files:**

**Supplementary Movie** The process of executing a distortion correction method using micro-scaled ruler calibration.

**Supplementary Text:**

1. **Scanning Sequence of MEMS-OR-PAM System**

Supplementary Fig. S1 shows the scanning sequence for the volumetric PA data acquisition for the MEMS-OR-PAM system. When the PA imaging is started, the MEMS mirror starts periodic (forward / reverse) motion, the pulsed laser continuously irradiates, and the mechanical scan starts in the Y-direction. At this time, the digitizer is triggered only in the half-cycle (forward direction) of the MEMS scan to acquire the PA signals. Therefore, the period $(T_{scan})$ and the frequency $(F_{scan})$ of the sinusoidal driving voltage to the MEMS mirror are determined by the following equation, which is adjusted to the time required to acquire the PA signal during the MEMS scan.

|  | $T_{scan}=\frac{1}{F_{scan}}=\frac{2\cdot N_{A-line}}{F_{laser}}$ |  |
| --- | --- | --- |

where $N_{A-line}$ is the number of PA signals (A-lines) acquired during a unidirectional scan (half cycle of the MEMS driving), and $F_{laser}$ is the PRF of the laser. The A-line acquisition starts after a delay time of $T_{scan}/4$ from the start timing of the MEMS driving cycle, and the 2D volumetric PA data for one B-mode image is acquired during $T_{scan}/2$. The Y-direction scan is performed by a mechanical stage (MicroPhotoAcoustic Inc., New York) in constant velocity linear motion throughout the imaging, and the motion velocity $V_{stage}$ is expressed as follows,

|  | $\boldsymbol{V}_{\boldsymbol{stage}}\boldsymbol{=}\boldsymbol{F}_{\boldsymbol{scan}}\boldsymbol{\cdot}\frac{\boldsymbol{R}_{\boldsymbol{y}}}{\boldsymbol{N}_{\boldsymbol{y}}}$ |  |
| --- | --- | --- |

where, $R_{y}$ is the imaging range in the Y-direction, and $N_{y}$ is the scanning point in the Y-direction (i.e., the number of B-mode acquired during the Y-direction scan). For example, when measuring an imaging area of 2 mm×2 mm with a step size of 400 points×400 points using a 10 kHz laser PRF, $T_{scan}=80 ms (F_{scan}=12.5 Hz)$, $V_{stage}=62.5 \mu m/s$, and the time for a 3D volumetric data acquisition is estimated to be about 32 seconds.

1. **Theoretical Spatial Resolution and Depth of Focus (DOF)**

**Lateral Resolution and DOF**

In OR-PAM, lateral resolution and DOF depend on the laser focus. Here, a simple model of the focusing optical system of developed MEMS-OR-PAM is shown in Supplementary Fig. S2. From this model, the spot diameter $D_{0}$ (1/e2 diameter), FWHM (1/2 diameter), and DOF at the focal position can be expressed by the following equations [1,2].

|  | $D_{0}=\frac{4\lambda F}{\pi D}M^{2}$ |  |
| --- | --- | --- |
|  | $FWHM=\frac{\sqrt{\ln2}}{\sqrt{2}}D_{0}\cong\frac{D_{0}}{1.7}$ |  |
|  | $DOF=\frac{2\pi{(D_{0}/2)}^{2}}{\lambda M^{2}}$ |  |

Where, λ is the laser wavelength (532 nm), $F$ is the focal length of the objective lens (60 mm), and $D$ is the beam diameter (10 mm) incident on the objective lens. $M^{2}$ is a beam quality factor that represents the degree of change in the beam from an ideal Gaussian beam. The beam quality when the laser is propagated by single-mode fiber approaches the ideal Gaussian beam ($M^{2}$≅1), and the beam quality by multimode fiber changes from the ideal Gaussian beam ($M^{2}$>1). The following equation can express the beam quality $M^{2}$ of the multimode fiber used in this study [3].

|  | $M^{2}=\frac{\pi\theta(d/2)}{\lambda}=2.95$ |  |
| --- | --- | --- |

Where, θ is the divergence angles of the fiber (0.1 rad in 0.1 NA), and $d$ is the core diameter of the multimode fiber (10 µm). From the Eq. (3-6), Theoretical lateral resolution (FWHM), and DOF can be calculated as follows.

|  | $Lateral Resolution \left( FWHM \right)=7.0 [\mu m]$ |  |
| --- | --- | --- |
|  | $DOF=165.3 [\mu m]$ |  |

**Axial Resolution**

The theoretical axial resolution depends on the frequency bandwidth of the ultrasound transducer, and it can be expressed as follows [4].

|  | $Axial Resolution=0.88\frac{c}{BW}=32.6 [\mu m]$ |  |
| --- | --- | --- |

Where, c is the speed of sound in water (=1480 m/s at 20 ℃), and BW is the bandwidth of the ultrasonic transducer (=40 MHz, 80% specific bandwidth in 50 MHz center frequency).

1. **Laser Safety in Microvascular Imaging In-Vivo**

In the PA imaging of living tissue, it is necessary to ensure the safety of laser energy density on the tissue surface. For this reason, MEMS-OR-PAM must consider the maximum permissible exposure for single laser pulse (MPE_SLP_) and for pulse train (MPE_TRAIN_), which are governed by American National Standards Institute (ANSI) [5,6,7].

MPE_SLP_ is defined as $2C_{A}{10}^{-2} J/{cm}^{2}=20 mJ/{cm}^{2}$ in the exposure duration of 10^-9^ to 10^-7^ s ($C_{A}$: wavelength correction factor, $C_{A}=1$ for visible wavelength range in 400-700 nm). Therefore, the laser pulse irradiated to the tissue surface must satisfy the following equation of laser energy density,

|  | $\frac{E_{pulse}}{\pi\left( {D_{surface}}/2 \right)^{2}}\leq20 [mJ/{cm}^{2}]$ |  |
| --- | --- | --- |

where $E_{pulse}$ is the pulse energy (400 nJ/pulse in microvascular imaging), and $D_{surface}$ is the effective laser beam diameter (1/e^2^ diameter) on the tissue surface. Here, to calculate the $D_{surface}$, we consider a laser irradiation model for living tissue as shown in Supplementary Fig. S3. When the experimental lateral resolution (FWHM diameter at the focal point) of MEMS-OR-PAM is 6 µm, the experimental numerical aperture (${NA}_{expt}$), which indicates the degree of the beam focusing, can be estimated as follows.

|  | ${NA}_{expt}=0.51\cdot\frac{\lambda}{{FWHM}_{focus}}\cong0.05$ |  |
| --- | --- | --- |

where λ is the laser wavelength (=532 nm), and ${FWHM}_{focus}$ is the FWHM diameter at the laser focus. ${NA}_{expt}$ is smaller than the NA of the objective lens because the optical aberration happened when the collimated laser beam with the large diameter irradiated on the objective lens and when the focused beam passed through the beam combiner. In the in-vivo finger microvascular imaging, the laser focus was located at a depth of about 300 µm from the skin surface. Therefore, the laser beam's FWHM diameter (${FWHM}_{surface}$) and effective diameter ($D_{focus}$) on the skin surface are calculated as follows.

|  | ${FWHM}_{surface}={FWHM}_{focus}+2\cdot{NA}_{expt}\cdot Depth=36 [\mu m]$ |  |
| --- | --- | --- |
|  | $D_{surface}=\frac{\sqrt{2}}{\sqrt{\ln2}}{\cdot FWHM}_{surface}\cong1.7{\cdot FWHM}_{surface}=61 [\mu m]$ |  |

Therefore, when the laser is irradiated at 400 nJ/pulse in the microvasculature imaging, the laser energy density on the skin surface is calculated as follows.

|  | $Energy Density=\frac{E_{pulse}}{\pi\left( {D_{surface}}/2 \right)^{2}}=13.7 [mJ/pulse]$ |  |
| --- | --- | --- |

Eq. (14) satisfies the MPE_SLP_ threshold in Eq. (10).

MPE_TRAIN_ is defined as $1.1C_{A}t^{0.25} J/{cm}^{2}=1.1t^{0.25} J/{cm}^{2}$, where t is the exposure duration in seconds. In microvascular imaging, the MEMS scanning was performed with the average pixel size of 5 µm and the laser PRF of 20 kHz (0.05 ms). Therefore, 12 (61 µm/5 µm) adjacent overlapped laser pulses are irradiated in one pixel, the summed exposure time is 0.6 ms (12×0.05 ms). As the result, MPE_TRAIN_ is calculated to be $172 mJ/{cm}^{2}$ and the MPE_SLP_ from the MPE_TRAIN_ is as follows.

|  | $\frac{{MPE}_{TRAIN}}{12}=14.3 [mJ/{cm}^{2}]$ |  |
| --- | --- | --- |

Therefore, the laser pulse energy used in this study fully met both${MPE}_{SLP}$ and ${MPE}_{TRAIN}$, and safety in-vivo PA imaging was achieved.

**Supplementary Figures:**


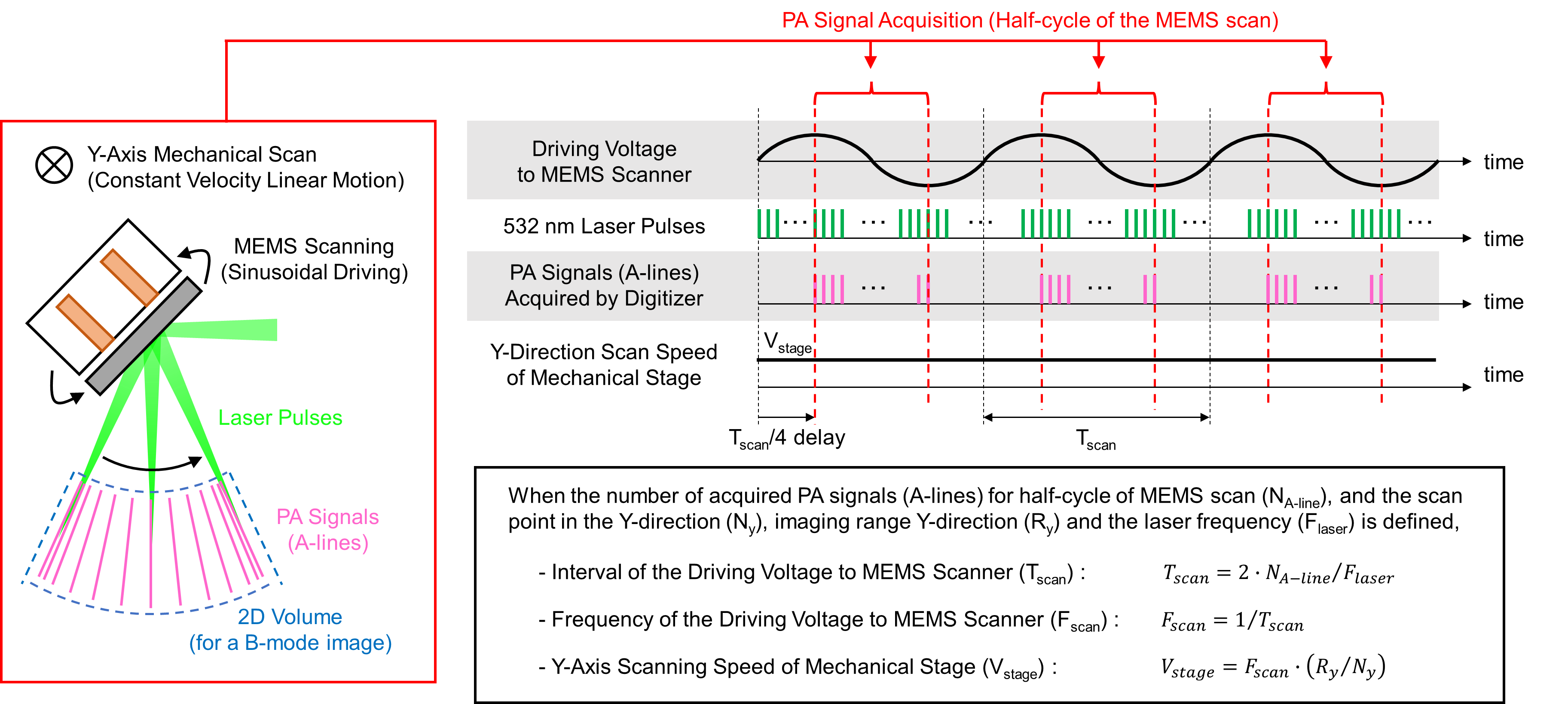


**Fig. S1** Scanning sequence of MEMS-OR-PAM system.


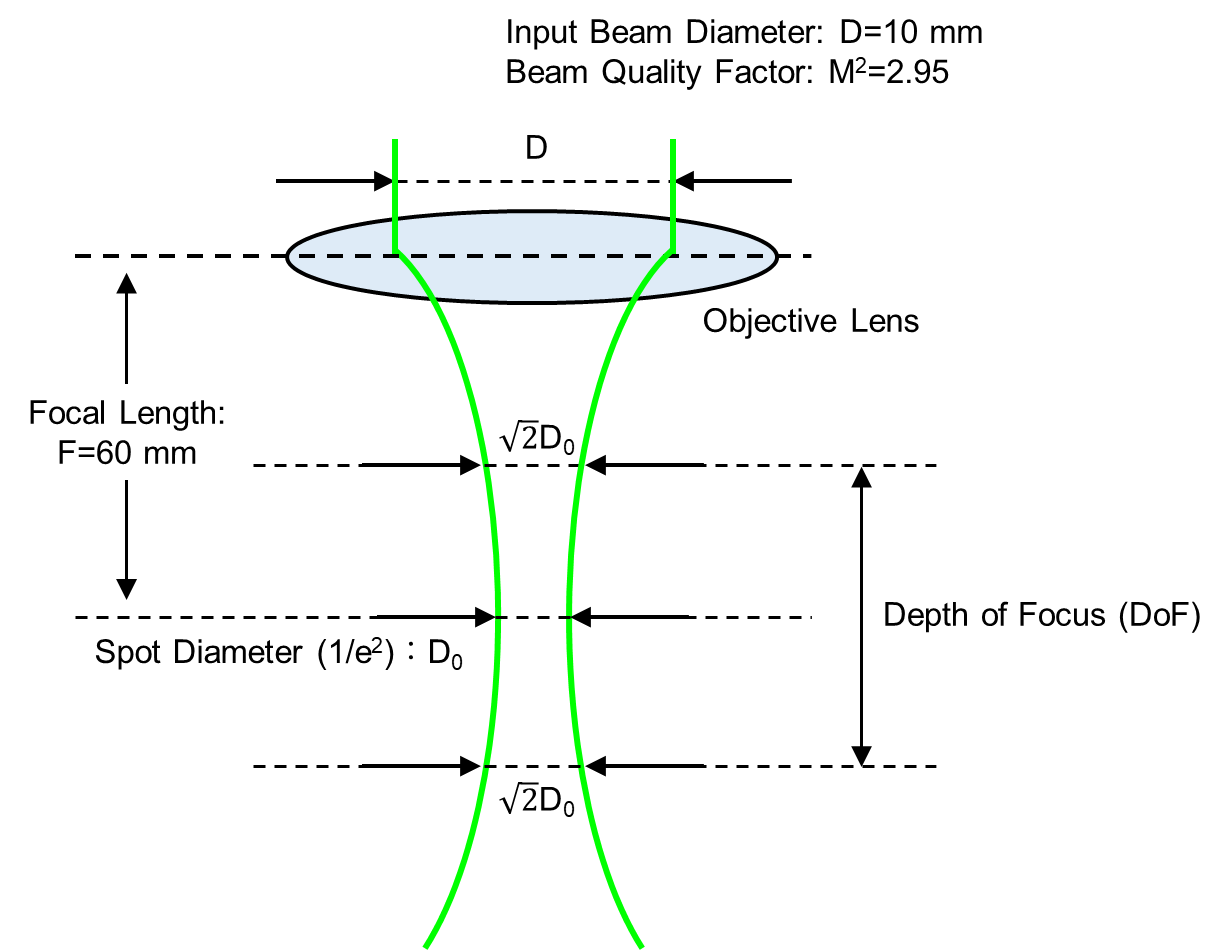


**Fig. S2.** Simple optical focusing model of MEMS-OR-PAM.


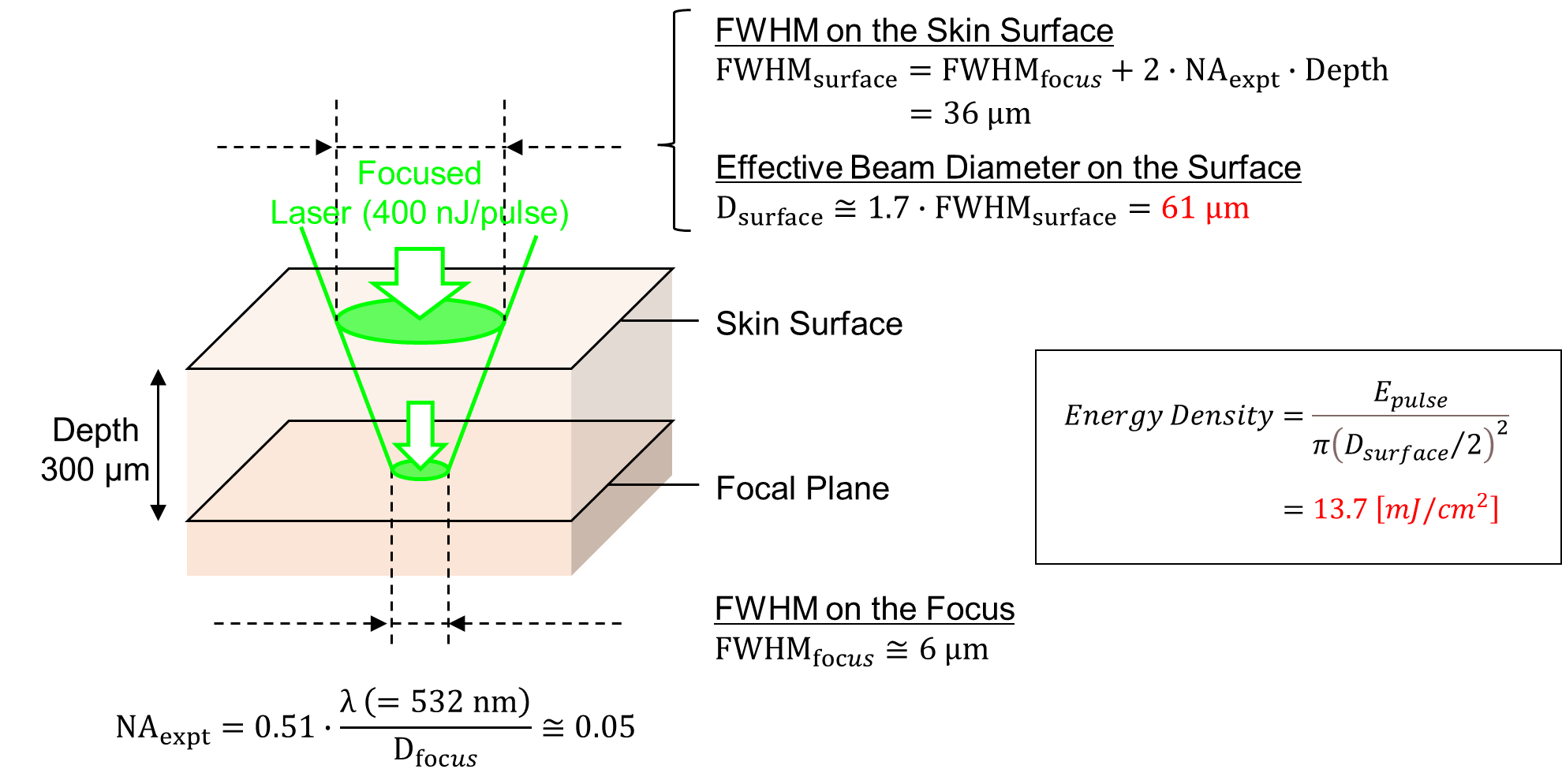


**Fig. S3.** Laser irradiation model for living tissue.


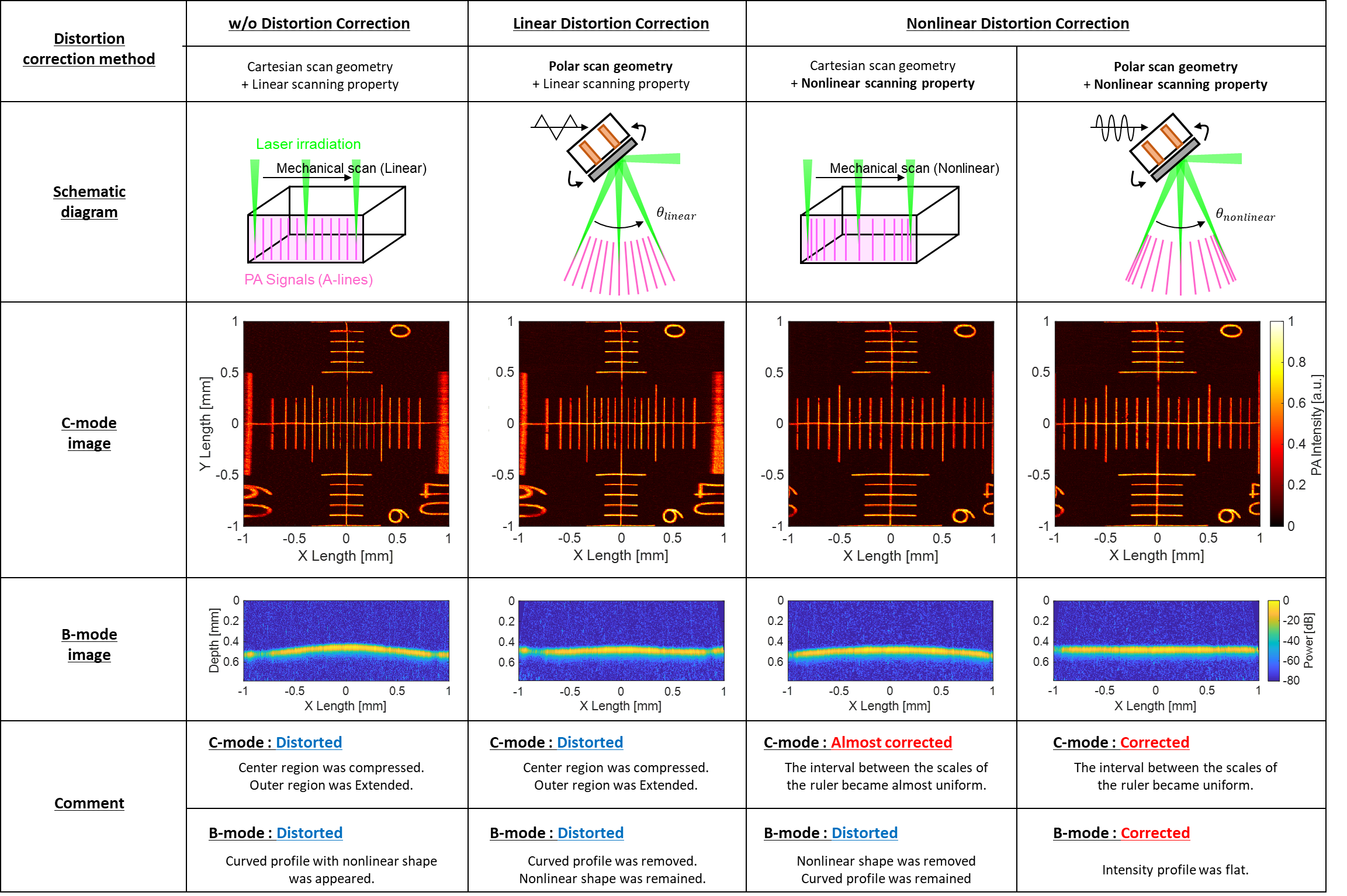


**Fig. S4** Comparison of all distortion correction approaches in ruler imaging.

**
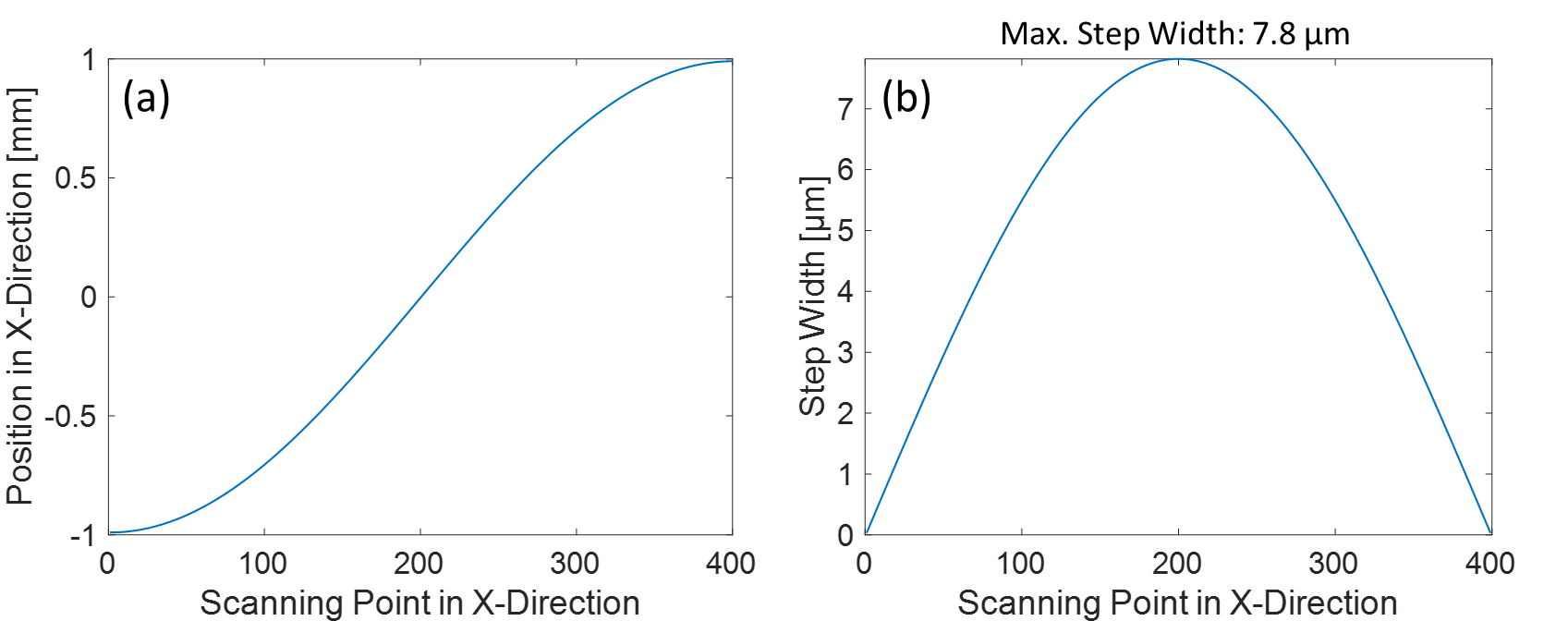
**

**Fig. S5** Scanning characteristics of a MEMS scan in the range of X＝2 mm at 400 points.

(a) Position in the X direction. (b) Property of the step width.

**Supplementary Reference**

1. Damask, J. N. Polarization Optics in Telecommunications. *Springer*, 221-223 (2004).
2. Siegman, A. E. Lasers. (University Science Books, Mill Valley, CA, 1986), Chap. 16.
3. ISO 11146-1:2005, Lasers and Laser-Related Equipment – Test Methods for Laser Beam Widths, Divergence Angles and Beam Propagation Ratios, International Organization for Standardization, Berlin Germany (2005).
4. Zhang, C., Maslov, K., Yao, J., Wang, L.V. In vivo photoacoustic microscopy with 7.6-µm axial resolution using a commercial 125-MHz ultrasonic transducer. *J. Biomed. Opt.* **17**, 116016 (2012).
5. American National Standard for Safe Use of Lasers (ANSI Z136.1–2000). New York, NY, USA (2000).
6. Baik, J.W., Kim, J.Y., Cho, S., Choi, S., Kim, J., Kim, C. Super Wide-field Photoacoustic Microscopy of Animals and Humans In Vivo. *IEEE Trans. Image Process.* **39**, 975-984 (2019).
7. Moothanchery, M. et. al, High-speed simultaneous multiscale photoacoustic microscopy, *J. of Biomed. Opt.* **24**, 086001 (2019).
